# Electronic–Structural Phase Correlations in Oxygen‐Deficient Hafnia Nanocrystals

**DOI:** 10.1002/smll.202508888

**Published:** 2025-11-20

**Authors:** Cristina Besleaga, Mihaela Botea, Catalin C. Negrila, Andrei Kuncser, Cosmin M. Istrate, Andrei Nitescu, George E. Stan, Swayam P. Sahoo, Bertrand Vilquin, Lucian Pintilie

**Affiliations:** ^1^ National Institute of Materials Physics Atomistilor 405A Magurele 077125 Romania; ^2^ Ecole Centrale de Lyon, INSA Lyon CNRS Universite Claude Bernard Lyon 1 Lyon 69130 France; ^3^ Laboratoire Nanotechnologies Nanosystèmes (LN2) ‐ CNRS UMI‐3463 Université de Sherbrooke Sherbrooke QC J1K 0A5 Canada

**Keywords:** FTIR spectroscopy, hafnia, low temperature phase transition, oxygen vacancies, pyroelectric effect

## Abstract

Layers of HfO_2_ and (Hf,Zr)O_2_ crystalline nano‐particles are synthesized via direct liquid injection atomic layer deposition, and a comprehensive set of structural, chemical, and electrical characterizations is employed to elucidate their phase composition and functional behavior. X‐ray photoelectron spectroscopy revealed a compositional contrast between the films: (Hf,Zr)O_2_ layers contained up to 45% stoichiometric oxide, while pure HfO_2_ films are dominated by sub‐oxides, especially under strongly reducing conditions, in which exclusively sub‐oxide phases and p‐type semiconducting behavior is revealed. Electrical measurements indicated room‐temperature stabilization of polar phases and tetragonal‐to‐orthorhombic phase transition with a Curie temperature near 200 K. FTIR spectroscopy confirmed the presence of tetragonal and orthorhombic HfO_2_ phases, providing insight into minor features observed ≈30° (2θ) in X‐ray diffraction patterns. Notably, devices incorporating an AlN interlayer demonstrated a significant enhancement in pyroelectric performance, suggesting this strategy to advance the pyroelectric performance of HfO_2_‐based materials, supporting their development for lead‐free sensor technologies.

## Graphical abstract

Layers of HfO_2_ nanoparticles are fabricated by DLI‐ALD. The resulting devices exhibit a pyroelectric response, contrary to expectations based on SAED and XRD results. A phase transition from orthorhombic to tetragonal phase (above 200 K) is revealed. The investigation indicates that the tetragonal phase transforms into a polar phase, a phenomenon conditioned by the presence of oxygen vacancies.

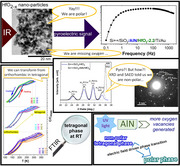

## 1 Introduction

Starting from 2011, following the initial report of ferroelectric behavior in hafnium oxide (HfO_2_)^[^ ^1^ ^]^ there has been a notable surge in research and review publications exploring HfO_2_ based materials. Its polar phases have sparked considerable interest due to the vast potential they offer for future applications such as pyroelectric energy harvesting, supercapacitors, memristors, and neuromorphic systems.

In the amorphous phase, hafnia finds widespread application as a high‐k dielectric in diverse semiconductor devices, such as Dynamic Random Access Memory capacitors and gate dielectrics for metal‐oxide‐semiconductor field‐effect transistors.^[^ ^2^ ^]^ Its high dielectric constant makes it particularly attractive for these applications, as it allows for the fabrication of capacitors and transistors with improved performance and reduced leakage current compared to traditional silicon dioxide (SiO_2_) dielectrics.

The well‐known HfO_2_ crystalline phases are the monoclinic (paraelectric) ground state (P21/c), the polar‐orthorhombic oIII‐phase (Pca21), the tetragonal t‐phase (P42/nmc), and the cubic c‐phase (Fm3m). The ferroelectric property of HfO_2_‐based materials was generally associated to Pca21 polar orthorhombic phase^[^ ^3^ ^]^, and only recently was evidenced for the polar tetragonal phase.^[^ ^4^ ^]^ Currently, the polymorphism of HfO_2_ is an open and vibrant research subject, of great interest for both fundamental and technological standpoints, as other new ferroelectric and antiferroelectric phases were observed.^[^ ^5^, ^6^ ^]^ Moreover, there were already reported experimental results that are not consistent with the conventionally considered phases.^[^ ^7^ ^]^

The source of this inconsistency could derive from the experimental limitation of the X‐ray diffraction (XRD) technique, which is by far the most utilized for hafnia‐phase identification.

Three theoretical studies,^[^ ^8^, ^9^, ^10^ ^]^ published in 2022, proposed Fourier transform infrared (FTIR) spectroscopy as an alternative powerful method for phase identification and discrimination in HfO_2_‐based materials.

In most of the cases, ferroelectricity was found in layers thinner than 10 nm,^[^ ^11^, ^12^ ^]^ but there are exceptions when hafnia‐based film was reported to be ferroelectric for thicknesses larger than 30 nm,^[^ ^13^ ^]^ of 390 nm^[^ ^14^ ^]^ and even up to 1 µm.^[^ ^15^ ^]^ In 2015, Materlik et al. employed a theoretical study determining that the window for a stable ferroelectric orthorhombic phase can occur between the tetragonal and monoclinic phases, as long as the grain sizes remain below 5 nm.^[^ ^16^ ^]^

Another intriguing yet less favorable phenomenon observed in ferroelectric HfO_2_ is wake‐up, characterized by a temporary rise in the switchable polarization over the initial 10^4^–10^5^ switching cycles.^[^ ^17^ ^]^ This occurrence has been attributed to the decrease in the non‐ferroelectric tetragonal interfacial layer, induced by the diminished concentration of oxygen vacancies (V_O_) within the layer.^[^ ^18^ ^]^ However, wake‐up was observed when used a reactive TiN electrode which could potentially increase the V_O_ concentration during repeated cycling.^[^ ^19^ ^]^ Consequently, the mechanisms behind these two phenomena, namely severe fatigue and wake‐up, as direct consequences of the V_O_ effect over the crystalline structure of hafnia, are poorly comprehended.

As highlighted in the recent article by F. Wunderwald, U. Schroeder, et al. ^[^ ^20^ ^]^ stress and strain in hafnia‐based materials are critical factors in stabilizing the polar phase. It is difficult to discriminate between stress and strain effects on polycrystalline thin films because stress is inducing elastic strain or plastic strain if dislocations or other extensive defects are created.

Stress arises from crystalline lattice distortion due to substrate/hafnia lattice mismatch or from post‐deposition thermal annealing due to different thermal expansion coefficients of substrate versus hafnia thin film.

Our study focuses on hafnia nanocrystals with a high density of oxygen vacancies.

Because hafnia layers are not compact/dense thin films but formed from agglomerated nanocrystals on a substrate, the stress parameter (as defined above) can be excluded from one of the causes of strain.

For discontinuous nanocrystal agglomerates, coalescence stress characterizing the Volmer‐Weber grown mechanism in thin films^[^ ^21^, ^22^ ^]^ can't be accounted for by strain.

Therefore, the nanoparticles' agglomeration layer's architecture can be used in accessing the other main sources for the strain presence in hafnia nanocrystals: grain boundaries or punctual defects.

Although the layer is composed by nanocrystal agglomeration and is not a compact/dense thin film, the grain size still impacts the intrinsic strain through the grain boundary density and associated defects. But since the investigated layers are composed of crystals with the same mean size, it is fair to assume that the differences in structural and electrical properties shown in this study are impacted by the presence of punctual defects whose concentration depends on the material processing conditions. For hafnia grown under oxygen‐poor conditions, the oxygen vacancies are the predominant punctual defects as it has the lowest formation energy.^[^ ^23^ ^]^

Interestingly, our investigation shows that oxygen‐deficient hafnia exhibits a pyroelectric effect, even though XRD analysis indicates a predominantly monoclinic HfO_2_ phase. Since the monoclinic phase is non‐polar, this observation is particularly intriguing, as such a phenomenon has not been previously reported.

To further probe the material's phase composition, we employed FTIR as an alternative to XRD, aiming to detect minor phases such as polar/non‐polar orthorhombic and tetragonal forms. These FTIR results were correlated with electrical measurements, leading to a comprehensive discussion of the unusual findings presented in this study.

Moreover, this study demonstrates that an AlN/HfO_2_ bilayer enhances the pyroelectric response of hafnia. This represents a simple yet effective strategy to advance the pyroelectric performance of HfO_2_‐based materials, supporting their development for lead‐free sensor technologies.

## 2 Results and Discussion

### 2.1 Results

HfO_2_ and (Hf,Zr)O_2_ layers were obtained by direct liquid injection (DLI) atomic layer deposition (ALD) following the procedure described in Experimental Method section. Deionized (DI) water, 0.5 M tetrakis(ethylmethylamido)hafnium(IV), (TEMA)Hf, in anhydrous toluene and 0.5 M solution in toluene of (TEMA)Hf and tetrakis(ethylmethylamido)zirconium(IV), (TEMA)Hf,Zr 1:1 mixt, were used as precursors for the deposition of HfO_2_ and (Hf,Zr)O_2_ thin films. The HfO_2_ layers were deposited at (TEMA)Hf / H_2_O mass flow ratios of 0.6, and 2.2 (**Table** **1**), and will be further denoted as HfO_2_‐0.6 and HfO_2_‐2.2, respectively. The (Hf,Zr)O_2_ layers synthesized at (TEMA)Hf,Zr / H_2_O mass flow ratios of 0.6, and 2.2, and, will be further referred as (Hf,Zr)O_2_‐0.6 and (Hf,Zr)O_2_‐2.2, respectively. The as‐deposited layers were amorphous; their crystallization was achieved by the application of a post‐deposition rapid thermal annealing at 600 °C/10 min, in nitrogen (1 atm), using heating and cooling rates of 5 °C min^−1^.

**Table 1:** Precursor mass flow rates and ratios used for the DLO‐ALD synthesis of the thin HfO_2_ and (Hf,Zr)O_2_ layers.

Sample	Flow rate (TEMA)Hf (g/min)	Flow rate H_2_O (g/min)	Flow ratio (TEMA)Hf/Flow H_2_O
HfO_2_‐0.6	0.43	0.75	0.57
HfO_2_‐2.2	1	0.45	2.22
(Hf,Zr)O_2_‐0.6	0.45	0.75	0.60
(Hf,Zr)O_2_‐2.2	1.10	0.49	2.24

TEM images characteristic to the *HfO_2_‐0.6*, *HfO_2_‐2.2*, (*Hf,Zr)O_2_‐0.6* and *(Hf,Zr)O_2_‐2.2* films are shown in Figure S1 (Supporting Information). Irrespective of material composition, the layers were composed of small, quasi‐spherical crystalline nanoparticles (NPs) with a diameter of ≈5 nm. Continuous films were evidenced in all cases. However, the films also elicited a fairly large thickness non‐uniformity, more marked, ≈50%, in the case of the highest water mass flow. The layers obtained at the higher (TEMA)M/ H_2_O mass flow ratio (where *M* is Hf or Hf:Zr) presented a decreased thickness non‐uniformity (of ≈30%). The non‐uniformity can be related to the water use as oxygen source.^[^ ^24^ ^]^

**Figure 1:**
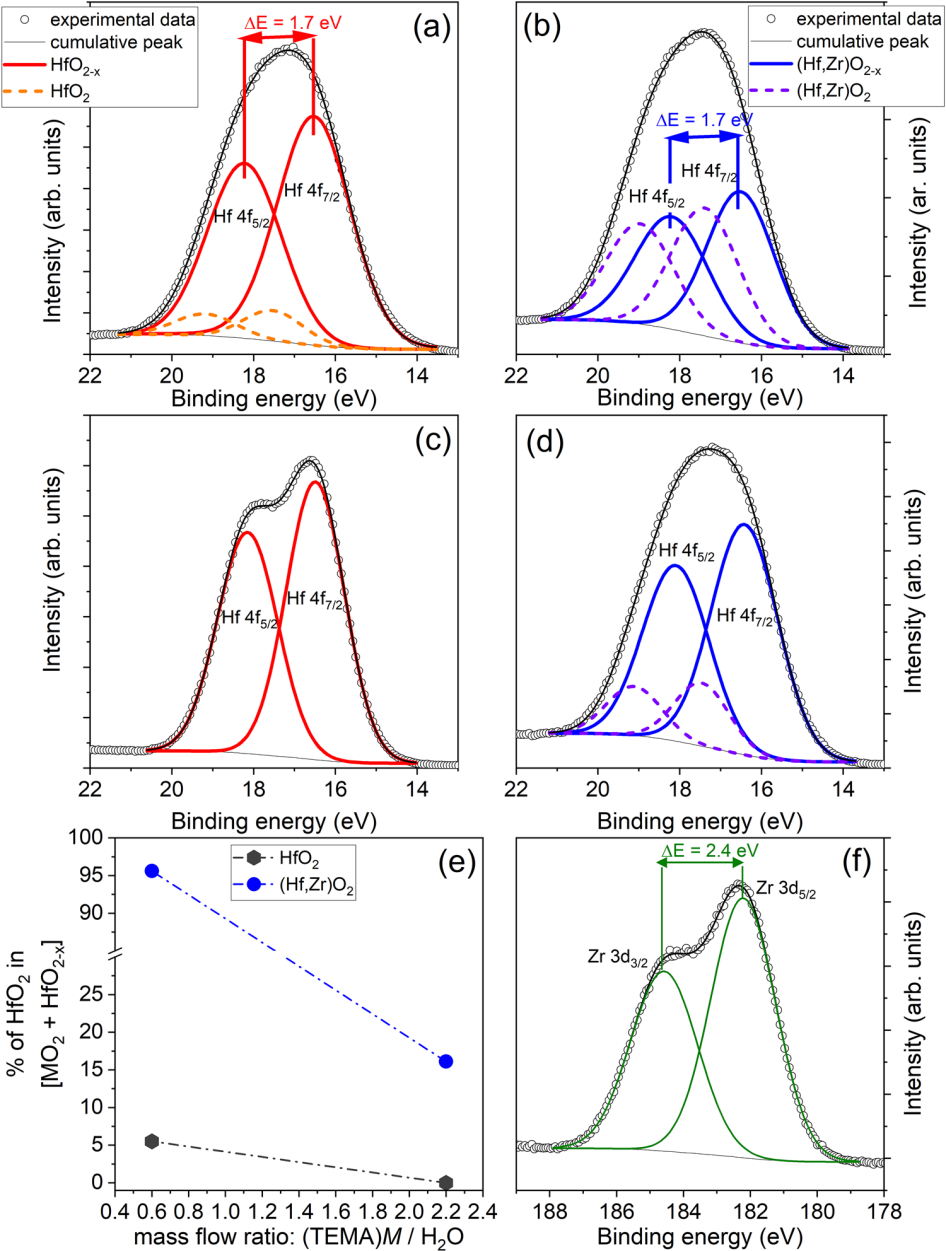
Hf 4f high‐resolution XPS spectra of a) HfO_2_‐0.6, b) (Hf,Zr)O_2_‐0.6, c) HfO_2_‐2.2, d) (Hf,Zr)O_2_‐2.2 e) Evolution of the oxide share versus (TEMA)M/ H_2_O flow ratio f) Zr 3d high‐resolution XPS spectra of (Hf,Zr)O_2_‐2.2.

The Hf 4f core electron spectra of the HfO_2_ and (Hf,Zr)O_2_ films are presented in **Figure** **1**a–d, while the Zr 3d core electron spectra of the (Hf,Zr)O_2_ films are presented in Figure 1f. The Hf:Zr atomic ratio for (Hf,Zr)O_2_‐0.6 is 1:1.1 and for (Hf,Zr)O_2_‐2.2 is 1:1.3. With the exception of HfO_2_‐2.2, all DLI‐ALD films presented the two pair of Hf 4 f 7/2 – Hf 4f 5/2 doublet peaks. The doublet peaks at ≈17.5 and ≈19 eV is associated with stoichiometric hafnium oxide,^[^ ^25^, ^26^, ^27^ ^]^ while the doublet at ≈16.5 and ≈18 eV can be attributed to hafnium sub‐oxides.^[^ ^25^, ^28^ ^]^

Thus, according to XPS, the obtained films are a mixture of stoichiometric and sub‐oxides hafnium oxides, the exception being made by HfO_2_‐2.2 which has no evidence of stoichiometric oxide. A significant difference in oxygen vacancy density can be depicted in the basis of the HfO_2‐x_ and HfO_2_ components ratio of the Hf 4f_7/2_ peak (Figure 3d). The Zr‐doped hafnia films exhibited larger percentages of stoichiometric oxide, up to 95.6%. The stoichiometric HfO_2_ phase stabilization role of Zr is thus suggested. The undoped hafnia ones were predominantly composed of sub‐oxides. In the case of the undoped hafnia layers, the largest HfO_2_ amount, of 5.5%, was obtained for the HfO_2_‐0.6, while no stoichiometric HfO_2_ presence was detected for HfO_2_‐2.2.

**Figure 2:**
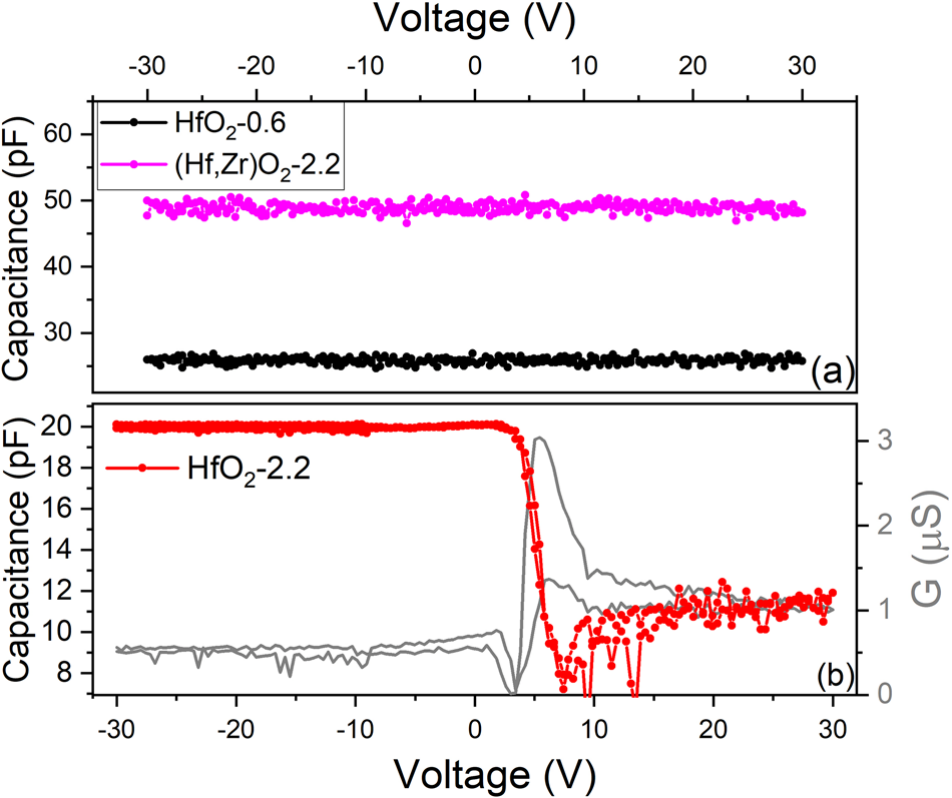
Capacitance versus Voltage characteristics at RT for a) Si/Mo/AlN/HfO_2_‐0.6 (black), Si/Mo/AlN/(Hf,Zr)O_2_‐2.2 (magenta), and b) Si/Mo/AlN/HfO_2_‐2.2 (red); 100 kHz, 0.5 V AC signal.

The electrical, C‐V and G‐V, measurements which revealed that the Si/Mo/AlN/HfO_2_‐2.2 device was the only one displaying a metal‐insulator‐semiconductor (MIS) characteristic, with the HfO_2_‐2.2 layer acting as a p‐type semiconductor (**Figure** **2**b). For all other investigated samples, with lower oxygen vacancy density (Figure 2a), the capacitance is constant with the applied voltage.

It was demonstrated that the presence of oxygen vacancies has a crucial role in molding the electrical properties in hafnia‐based materials and when in large concentrations are known to induce p‐type conduction in hafnium oxide‐based materials.^[^ ^29^ ^]^ Moreover, the poorly comprehended mechanisms behind ferroelectricity, the severe fatigue and wake‐up phenomena, are consequences of the V_O_.^[^ ^17^ ^]^

Therefore, we further focused on the material with higher concentration on oxygen vacancies, HfO_2_‐2.2, and to evidence the impact of the substrate nature over the hafnia characteristics, HfO_2_‐2.2 was deposited on both oxide (SiO_2_) and nitride (AlN) layers.

As in the case of Si/Mo/AlN/HfO_2_‐2.2, the recorded C‐V curves for Si++/SiO_2_/HfO_2_‐2.2 are specific to a MOS device where the HfO_2_ plays the role of a p‐type semiconductor (Figure S2, Supporting Information). Thus, the existence of a significant V_O_ concentration in HfO_2_‐2.2 layers is supported when both AlN and SiO_2_ substrate is used.

**Figure 3:**
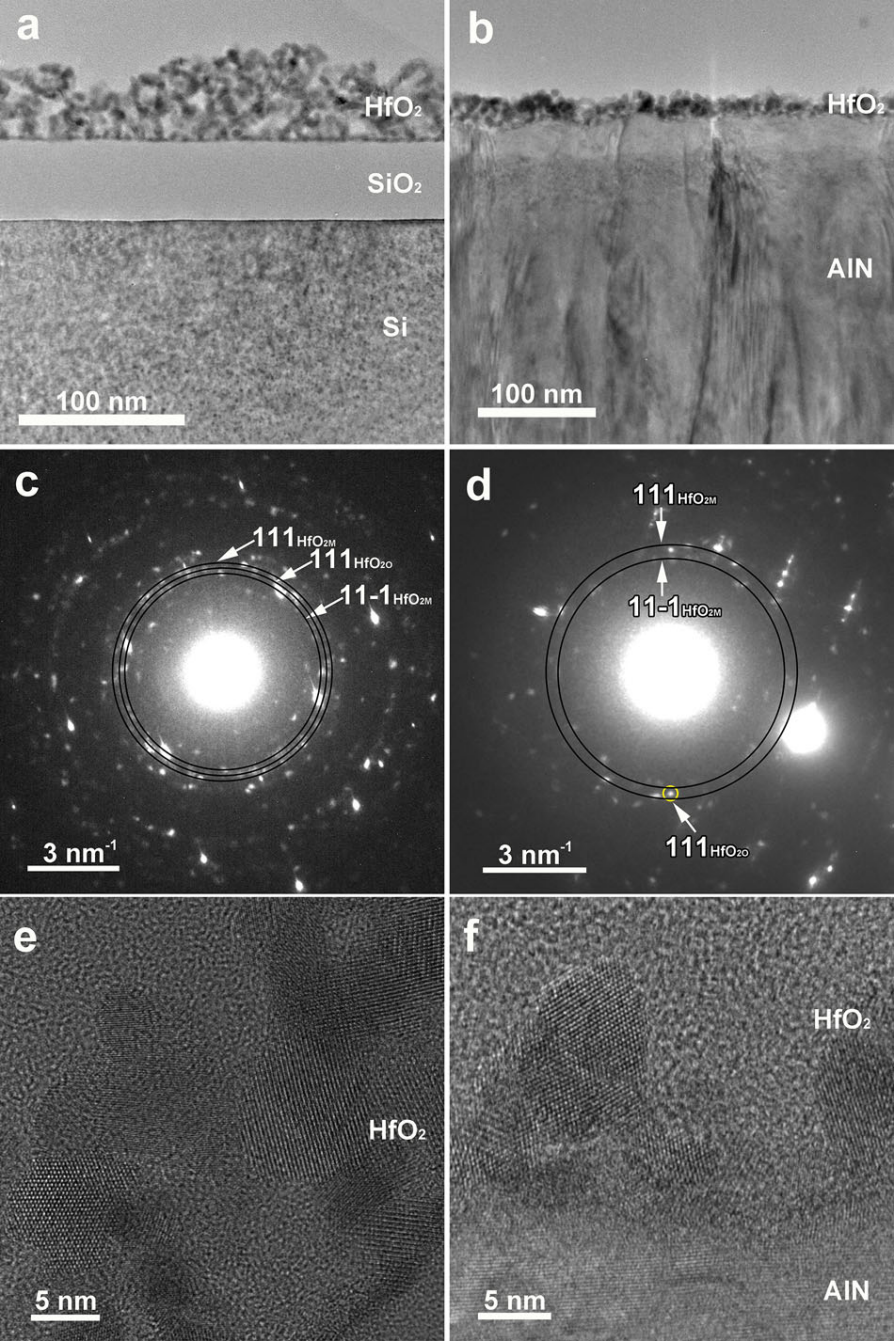
TEM image at low magnification and the corresponding SAED pattern of the a, c) Si/SiO_2_/ HfO_2_‐2.2 and b, d) Si/SiO_2_/AlN/HfO_2_‐2.2 heterostructures; TEM image at high magnification of the e) Si/SiO_2_/ HfO_2_‐2.2 and f) Si/SiO_2_/AlN/ HfO_2_‐2.2 heterostructures.

A non‐uniformity in terms of HfO_2_ layer thickness was evidenced, which ranged from 15 to 35 nm across the substrate, irrespective of type (SiO_2_ or AlN) interlayer (**Figure** **3**). In both cases, the HfO_2_ layer was composed of packed spherical, slightly elongated or rectangular with rounded corners nanoparticles (NPs) with sizes between 5 and 15 nm.

TEM/SAED results (Figure 3) indicated that in the case of both specimens, HfO_2_ is undoubtedly crystallized in the monoclinic system (Figures S3 and S4, Supporting Information), although evidences of traces of a tetragonal‐ or orthorhombic‐type phase cannot be excluded in the case of hafnia deposited on AlN, as hinted by a faint SAED spot which could be assigned either to (111) orthorhombic phase, either to (101) tetragonal phase.

**Figure 4:**
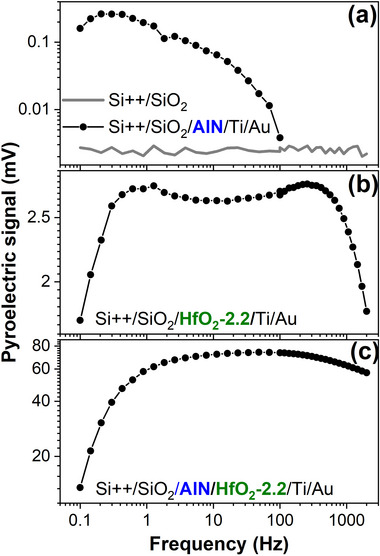
The frequency dependence of the pyroelectric signal for a) Si++/SiO_2_ (gray line), Si++/SiO_2_/AlN, b) Si++/SiO_2_/HfO_2_‐2.2, and c) Si++/SiO_2_/AlN/HfO_2_‐2.2 devices.

The HfO_2_‐2.2‐based devices showed a pyroelectric response (**Figure** **4**), contrary to what could be expected, considering the SAED results (Figure 3c,d) which indicated that the obtained HfO_2_ is crystallized in the monoclinic system, which is a non‐polar‐type phase whose presence can lead to the inhibition of the pyroelectric response.^[^ ^30^ ^]^ Please note that the reference device, i.e., Si++/SiO_2_/Ti/Au, showed no pyroelectric effect.

Unquestionably, the existence of a pyroelectric effect in HfO_2_ layers implies the presence of polar crystalline phases. No such polar phases were detected at the sensitivity limit of the employed SAED method for SiO_2_/HfO_2_‐2.2, and only a faint spot suggesting the presence of either to (111) orthorhombic phase, either to (101) tetragonal phase, for AlN/HfO_2_‐2.2.

**Figure 5:**
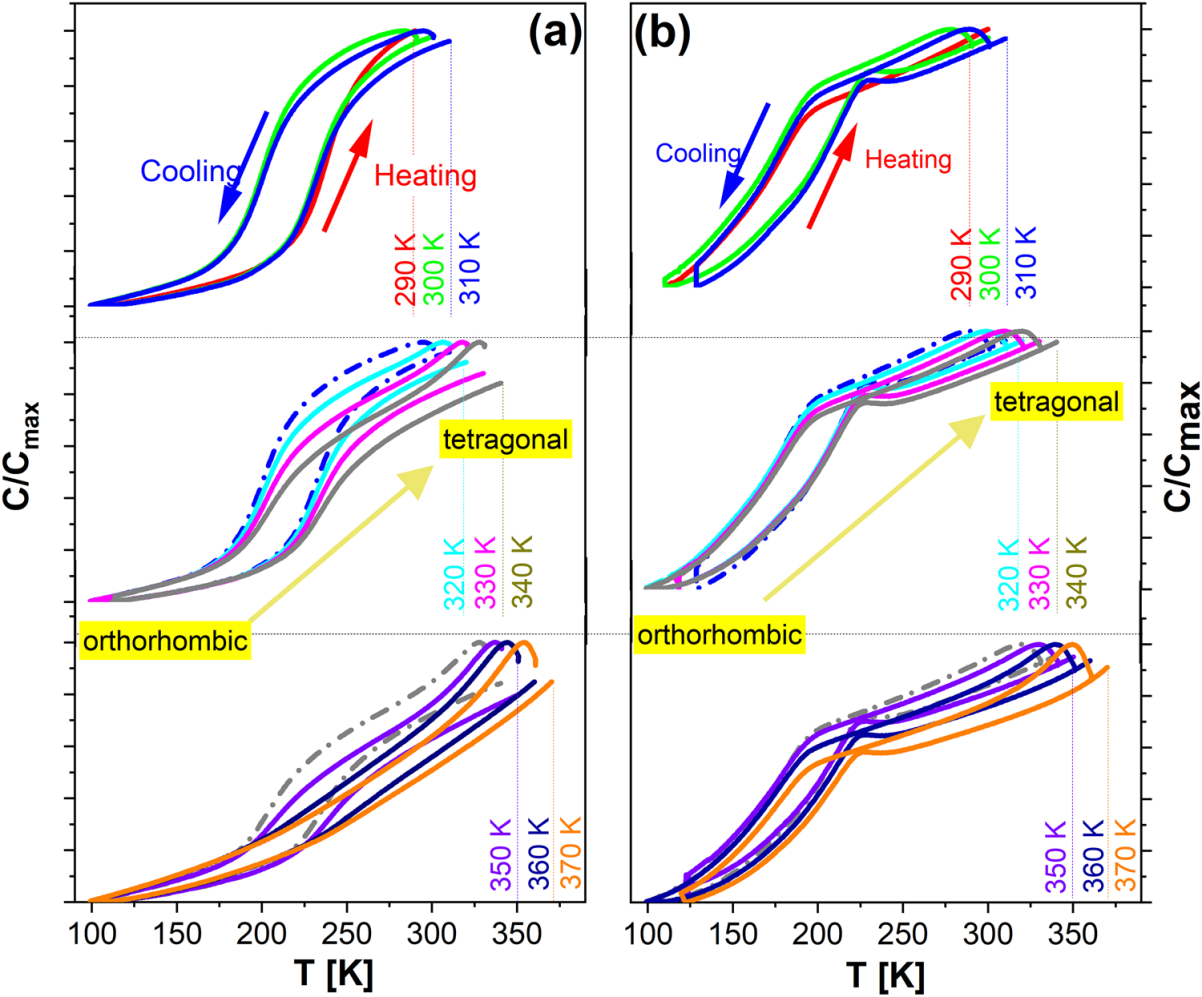
Capacitance versus Temperature measured at 100 kHz with AC signal of 0.5 V for a) Si++/SiO_2_/ HfO_2_‐2.2 and b) Si++/SiO_2_/AlN/ HfO_2_‐2.2 devices; 100 kHz, 0.5 V AC signal.

In order to shed light on this apparent peculiarity, Capacitance versus Temperature measurements were performed on the same devices, disclosing a phase transition ≈200 K (**Figure** **5**), which can be attributed to a tetragonal‐to‐orthorhombic phase transition,^[^ ^31^ ^]^ with orthorhombic as low‐temperature phase. The transition from a lower symmetric monoclinic P21/c phase to stable orthorhombic Pca21 or tetragonal P42/nmc phases can be excluded, due to the low thermodynamic energy of the monoclinic phase.^[^ ^32^ ^]^

Hoffmann et al. reported a high pyroelectric coefficient (≈1 mCm^−2^K^−1^) for Si:HfO_2_ (4.3 mol% Si) thin films, exhibiting tetragonal‐to‐orthorhombic phase transition ≈200 K,^[^ ^33^ ^]^ in agreement with our findings. In their study, Hoffmann et al. calculated the pyroelectric coefficients from the remanent polarization and showed that the maximum value corresponds to the Curie temperature, followed by a slow decrease toward RT. Hoffmann et al. concluded that the high pyroelectric coefficient derives mostly from the orthorhombic‐to‐tetragonal transition, rather than from the proper pyroelectric behavior, which has an estimated contribution of ≈2% from the total recorded pyroelectric coefficient.^[^ ^33^ ^]^

Hoffmann et al. theory could not explain the results depicted in Figure 4 as the measurements were done at room temperature, without cooling and then heating the sample, so without previously passing through a structural phase transition.

In the framework of this study, it was observed that, for the AlN/HfO_2_‐2.2 system, the tetragonal‐to‐orthorhombic phase transition was maintained even after thermal annealing in dark vacuum up to 370 K (Figure 5b). Remarkably, for the SiO_2_/HfO_2_‐2.2 configuration, the phase transition could not be detected if the sample was subjected to heating at temperatures in excess of 360 K (Figure 5a); in order to reproduce the tetragonal‐to‐orthorhombic phase transition, the sample was irradiated with UV light prior measurement.

The UV irradiation is known to induce oxygen vacancies in oxide materials.^[^ ^34^ ^]^ For monoclinic HfO_2_, the most stable configuration is the double‐charged V_O_^++^, a defect state situated at 1 eV below the conduction band.^[^ ^35^ ^]^ The presence of V_O_^++^ is owned to the photoionization of existing and newly created oxygen vacancies, a process accompanied by lattice relaxation.^[^ ^34^ ^]^ Furthermore, a large V_O_^++^ concentration was found to promote the tetragonal phase formation at RT.^[^ ^35^ ^]^ When the activation energy is provided by adequate thermal heating, this defect becomes electrical neutral by charge de‐trapping. In the absence of electrical charged V_O_^++^ defects, the tetragonal phase is not formed and, consequently, the tetragonal‐to‐orthorhombic phase transition no longer occurs. Contrariwise to *HfO_2_‐2.2* deposited on SiO_2_, where the V_O_^++^ defects are extrinsically formed by exposing the device to UV light, in the case of the AlN/HfO_2_‐2.2 system the formation of oxygen vacancies in HfO_2_ has an additional source caused by the high affinity for oxygen of the AlN layer, which can be further enhanced by nitrogen deficiency.^[^ ^36^, ^37^ ^]^ The phenomena of oxygen vacancies formation due to the chemical interaction between metal nitride and hafnia has been studied extensively^[^ ^38^ ^]^ and it is assumed that is one of the causes for tetragonal and orthorhombic phase stabilization.

The large cooling‐heating temperature hysteresis (Δ*T* = 30 K) implies a significant lattice deformation when first‐order tetragonal‐to‐orthorhombic transition takes place.^[^ ^39^ ^]^

**Figure 6:**
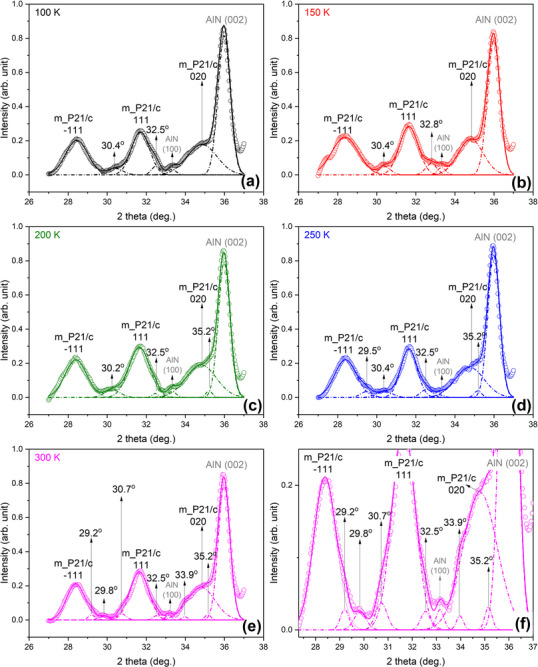
GIXRD patterns of the HfO_2_‐2.2 on AlN at various temperatures: a) 100 K, b) 150 K, c) 200 K, d) 250 K, e) 300 K and f) zoom‐in of the XRD pattern measured at 300 K.

At first glance, the GIXRD investigations revealed the monoclinic (P21/c system) as the dominant phase in the obtained HfO_2_ films but the peaks fitting of GIXRD patterns unveiled the presence of an additional crystalline phase, characterized by a reflection centered at 2θ ∼30°, 32.5°, and 35.2°, disclosed at all measurement temperatures (**Figure** **6**). The peak situated at 30.7° can be attributed to both orthorhombic Pbca (reflexion on 211 plane) or to cubic phase (space group Fm‐3m, reflection on 111 plane). The presence of cubic phase is also suggested by the XRD reflection at 2θ = 35.2° (002 plane). It was previously demonstrated^[^ ^29^ ^]^ that reducing the oxidation conditions, the monoclinic reflection is gradually reduced in intensity and accompanied by the appearance of cubic phase, thus the presence of cubic phase is plausible in hafnia with large V_O_ density. The presence of non‐polar orthorhombic Pmnb phase is observed for all temperature range being indicated by the peak ≈32.5° (reflection on 020 crystalline plane, while the peaks at ≈ 29.5° and ≈33.9°, observed above 200 K, can be associated to the tetragonal phase (space group P42/nmc) 101 and 110 planes respectively, thereby confirming the assumptions made concerning the results presented in Figure 5.

One can notice when comparing the patterns registered at 100 K and at 300 K, below and above Curie temperature, it can be observed that significant changes takes place ≈2θ ∼30°. The phase association (indexing) of this particular reflection was found to be highly problematic, since the orthorhombic (Pca21 system) is known to be virtually indistinguishable from the non‐polar, tetragonal P42/nmc^[^ ^35^ ^]^ and cubic phase (as shown in Table S1, Supporting Information) moreover when the analyzed hafnia is in thin film form which is known to be highly affected by the stress effects translating in XRD peaks shifting from their theoretical or bulk corresponding positions. In our case, the layers consist from agglomeration of nano‐grains of hafnia, and, thus, the stress accumulation in the layer can be considered to be null.

Overall, the signal originated from all these phases is minor compared to the one corresponding to the monoclinic phase, and the peaks deconvolution presented in Figure 6 can be rightly judged as not convincing for the presence of other crystalline phases.

**Figure 7:**
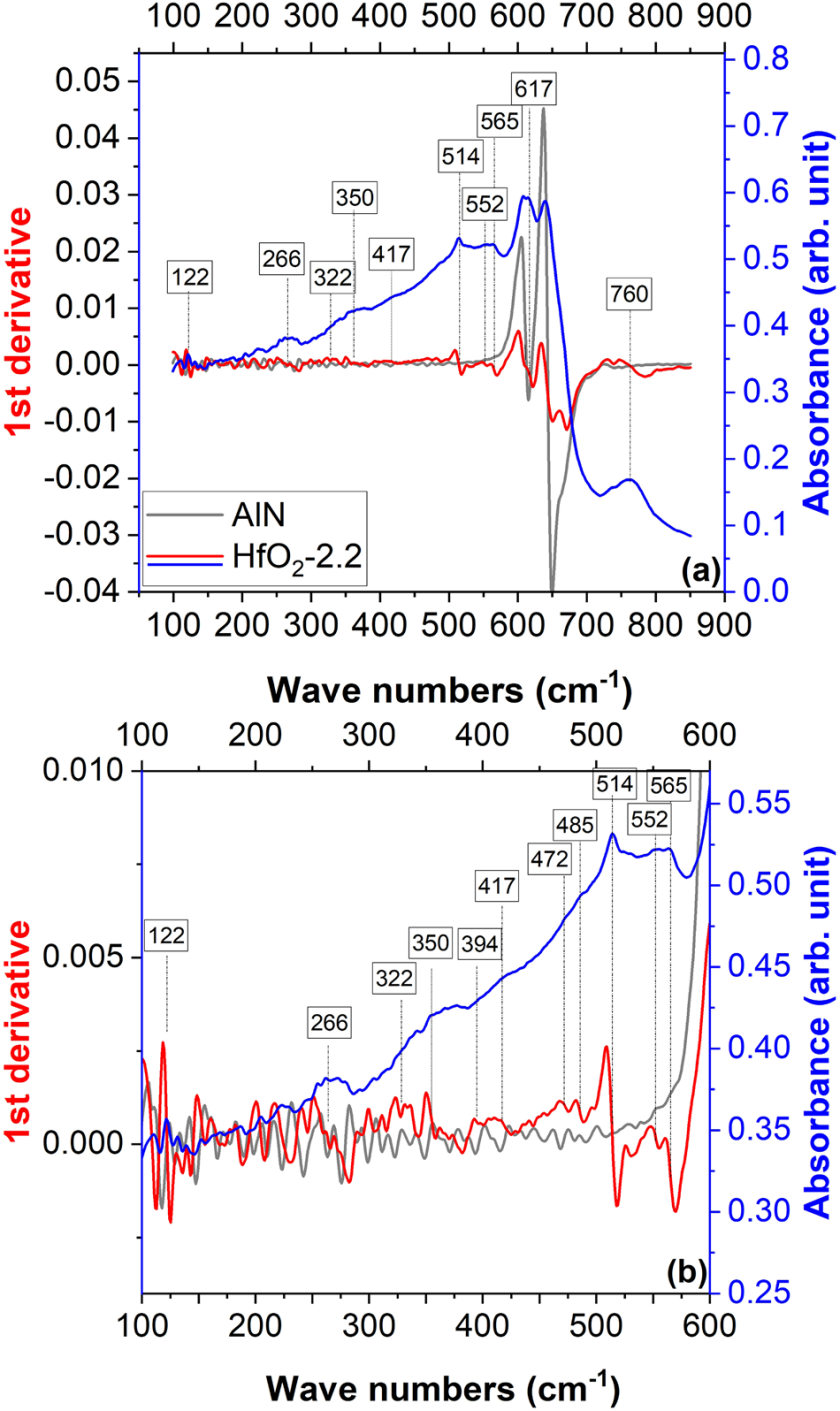
The FTIR‐ATR spectra of the HfO_2_‐2.2 and AlN; a) investigation domain: 100–900 cm^−1^, b) zoom‐in: 100–600 cm^−1^; absorbance (blue line) and first derivative (red line for HfO_2_‐2.2 and gray line for AlN).

Aiming to clarify the origin of the yet‐to‐be‐identified minor phases, FTIR spectra were collected in the far‐infrared region, where the lattice vibrational features of most metallic oxides are exhibited. In fact, the FTIR spectroscopy was theoretically suggested as a powerful tool for the phase identification in HfO_2_‐based thin films^[^ ^8^, ^9^ ^]^ and, very recently, the nano‐FTIR method was used to differentiate between the ferroelectric phase and other metastable phases.^[^ ^40^ ^]^ The FTIR spectra collected over an extended wave numbers domain of 100 – 850 cm^−1^, are presented in **Figure** **7**.

In 2022, Fan et al. published a theoretical study were the phonon band structures for the P21/c (monoclinic), Pca21 (polar‐orthorhombic oIII‐phase), Pbca (antipolar orthorhombic oI phase), P42/nmc (tetragonal), and Fm3m (cubic) HfO_2_ phases were computed using PBEsol density functional.^[^ ^8^ ^]^ Moreover, using ab initio simulations, Kersch et al. ^[^ ^9^ ^]^ identified the IR fingerprints of the HfO_2_ and ZrO_2_. These two compressive theoretical studies were considered for the association of the FTIR peaks to vibration modes specific to hafnia phases (Supporting Information).

In their empirical study, S.T. Jaszewski et al. ^[^ ^40^ ^]^ identified a clear signature of the polar orthorhombic phase at 775 cm^−1^, a vibration that was not observed in our samples. Instead, our FTIR analysis revealed a vibration mode at ≈322 cm^−1^ which can be attributed only to the tetragonal phase. This observation corroborates the XRD deconvolution results, which indicated the presence of the 137/P4_2_nmc structure above 200 K, thereby complementing the interpretation of the capacitance–temperature measurements.

### 2.2 Discussion

The XPS investigation showed that the density of oxygen vacancies was significantly reduced by Zr‐doping, while keeping unchanged all the deposition parameters. This was enhanced at a (TEMA)*M*/H_2_O mass flow ratio of 0.6. These findings are found good agreement with previous reports^[^ ^41^ ^]^, including with Pavoni et al.,^[^ ^42^ ^]^ which indicated that the defect formation energy is increased by Zr incorporation, i.e., (*i*) in monoclinic systems (P21/c), from 0.17 eV for HfO_2_ to 0.21 eV for Hf_0.5_Zr_0.5_O_2_, and in (*ii*) orthorhombic systems (Pca21), from 0.37 eV for HfO_2_ to 0.82 eV for Hf_0.5_Zr_0.5_O_2_. Thus, it is suggested^[^ ^42^ ^]^ that the monoclinic phase has a higher susceptibility to form oxygen vacancies, and this characteristic gradually attenuates when the amount of incorporated Zr is increased.

It is known that, for thin films, the presence of Zr in HfO_2_ leads to the formation of the polar orthorhombic phase, which is closely related to the ferroelectricity property.^[^ ^43^, ^44^, ^45^, ^46^ ^]^ At the same time, the GIXRD data revealed no differences between HfO_2_‐2.2 and (Hf,Zr)O_2_‐2.2 layers (Figure S5, Supporting Information). Thus, for the layers consisting of an agglomeration of ≈5 nm grains (Figure S1, Supporting Information), which are no affected by the macrostrain, the presence of Zr did not enhance the growth of a polar phase.

When dealing with compact thin layers, like (Hf,Zr)O_2_ deposited by RF‐magnetron sputtering,^[^ ^47^ ^]^ the substrate‐to‐layer stress impacts the structural arrangement and favors the formation of polar orthorhombic phase (Figure S6, Supporting Information). The increase of the compact film thickness leads to a stress release translated in lower strain values and consequently to an increased monoclinic phase ratio, as showed in Figure S7 (Supporting Information).

Interestingly, for (Hf,Zr)O_2_ films deposited by RF‐magnetron sputtering, the lattice strain relaxation and consequently the grow of monoclinic phase is accompanied by the tetragonal phase (marked by XRD reflection at ≈29.5° and FTIR vibration mode at 322 cm^−1^ (Figure S8, Supporting Information), as in the case of HfO_2_‐2.2 layers.

For HfO_2_‐2.2, the presence of the tetragonal phase at room temperature, which is not known as a polar phase, was demonstrated by capacitance versus temperature, XRD, and FTIR investigations. In the same time, in both XRD and FTIR analyses, the minor presence of polar orthorhombic phase is suggested but not undisputed. Thus, the structural analysis could not explain the pyroelectric signal measured in the case of HfO_2_‐2.2.

However, the electric field driven phase transition was reported for HfO_2_‐based materials;^[^ ^30^, ^33^, ^48^ ^]^ it is stated that the non‐polar phase transforms into the polar‐orthorhombic phase when oxygen vacancies migrate, driven by an electric field, from the interface into the HfO_2_ bulk region.^[^ ^17^, ^49^ ^]^ Moreover, the tetragonal phase is only a few meV/f.u. Close to the ferroelectric (orthorhombic) phase, such that an electric field is sufficient to induce its stability.^[^ ^35^ ^]^ The validation of the ferroelectric phase existence at RT was not possible through remanent polarization measurements for the HfO_2_‐2.2 layers. This can be due to the semiconductor nature of the investigated HfO_2_‐2.2. Even if the ferroelectric phase and conductivity coexist, in the case of a semiconductor material, an external electric field induces a flow of electric current which makes the switching of the ferroelectric displacements difficult.^[^ ^50^ ^]^

The pyroelectric signal for Si++/SiO_2_/(Hf,Zr)O_2_‐2.2 is significantly lower than the Si++/SiO_2_/HfO_2_‐2.2 one, Figure S9 (Supporting Information). This result, together with the previously mentioned XPS and XRD findings, supports the idea that the significantly larger pyroelectric signal in HfO_2_‐2.2 originates from the higher density of oxygen vacancies in this material.

Furthermore, the AlN/HfO_2_‐2.2 devices exhibited a higher pyroelectric signal with respect to the SiO_2_/HfO_2_‐2.2 ones. In low‐frequency pyroelectric applications, this remarkable phenomenon can originate from the strain induced in the film‐substrate system.^[^ ^51^ ^]^ In most of the cases, this strain stems from the considerably different coefficients of thermal expansion (CTE) of the two adjoining materials, but this theory does not fit our results, since AlN and HfO_2_ have similar CTEs, i.e., AlN ≈ 5.3 × 10^−6^ K^−1^^[^ ^52^ ^]^ and HfO_2_ ≈ 6 × 10^−6^ K^−1^.^[^ ^53^ ^]^ Instead, the enhancement of the pyroelectricity can be related to the polar nature of AlN. When the HfO_2_ thin layer is deposited directly on the surface of the AlN, a built‐in electric field is induced, whose direction depends on the polar AlN surface termination. Such an internal electrical field is promoting the transition from tetragonal to polar orthorhombic phase,^[^ ^35^, ^54^ ^]^ hence the enhanced pyroelectric effect in AlN/HfO_2_‐based devices. The AlN/HfO_2_ system was previously analyzed by Ye et al. ^[^ ^55^ ^]^, Reid et al.,^[^ ^56^ ^]^ Augustin et al.,^[^ ^57^ ^]^ Binh Do et al. ^[^ ^58^ ^]^ but, to the best of our knowledge, no previous studies have underlined the capacity of AlN/HfO_2_ bi‐layers to improve the overall performance of corresponding pyroelectric devices. Thus, the data reported here reveal a new and convenient method to push forward the pyroelectric performance of HfO_2_‐based devices toward lead‐free sensor technology.

## 3 Conclusion

Layers of HfO_2_ and (Hf,Zr)O_2_ crystalline nano‐particles were fabricated at 150 °C by DLI‐ALD and post‐deposition annealed at 600 °C. A suite of physical‐chemical and electrical measurements was applied to gauge their crystalline phase origin.

The XPS results showed that the obtained films are a mixture of stoichiometric and sub‐oxides. The (Hf,Zr)O_2_ thin film samples exhibited a larger percentage of stoichiometric oxide, up to 45%, while the pure hafnia layers were predominantly composed of sub‐oxides, with the extreme case of films deposited at the highest reducing working atmosphere (i.e., HfO_2_‐2.2) for which no stoichiometric oxide phase was evidenced. The same material, HfO_2_‐2.2, was the only one eliciting semiconductor (p‐type) behavior, as demonstrated by the C‐V measurements.

The capacitance versus temperature measurements revealed a phase transition at ∼ 200 K assigned to the transition from orthorhombic (at low temperatures) to the tetragonal phase (above 200 K). Moreover, FTIR spectroscopy analyses allowed to reveal with a definite degree of certainty the presence of tetragonal and orthorhombic HfO_2_ phases. These investigations shed light over the crystalline phase corresponding to the GIXRD minor peaks at ≈30° (2θ), evidenced by pattern deconvolution.

The pyroelectric tests emphasized the formation of polar phases at room temperature, suggesting that the non‐polar tetragonal phase transforms into a polar phase, a phenomenon conditioned by the presence of oxygen vacancies.

Remarkably, the pyroelectric signal recorded for Si++/SiO_2_/AlN/HfO_2_/Ti/Au configuration is larger than the one corresponding to Si++/SiO_2_/HfO_2_/Ti/Au. These results could pave the road toward the development of more market‐competitive lead‐free pyroelectric sensors.

## 4 Experimental Section

### Materials

**Figure 8:**
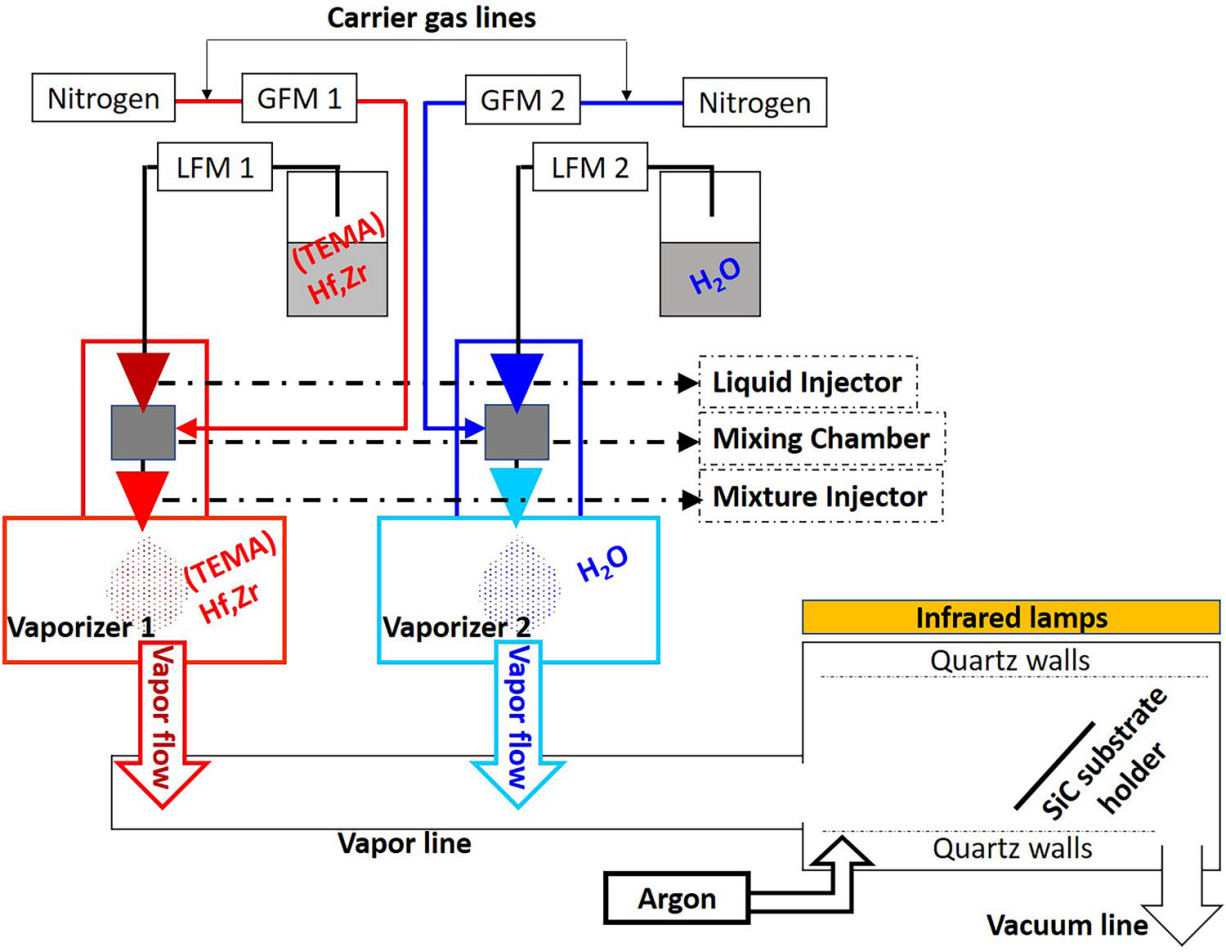
Schematic of the employed DLI‐ALD system.

HfO_2_ and (Hf,Zr)O_2_ layers were obtained by direct liquid injection (DLI) atomic layer deposition (ALD) using an Annealsys MC‐050DLI‐CVD/ALD & RTP system (a sketch of the deposition system is presented in **Figure** **8**). The DLI‐ALD method enables the utilization of thermally unstable, viscous, and low vapor pressure chemical precursors. Moreover, DLI‐ALD permits the use of both liquid and solid precursors dissolved in an organic solvent, being thus, a versatile method allowing the facile change of the composition and concentration of the precursors.

Deionized (DI) water, 0.5 M tetrakis(ethylmethylamido)hafnium(IV), (TEMA)Hf, in anhydrous toluene and 0.5 M solution in toluene of (TEMA)Hf and tetrakis(ethylmethylamido)zirconium(IV), (TEMA)Hf,Zr 1:1 mixt, were used as precursors for the deposition of HfO_2_ and (Hf,Zr)O_2_ thin films. The precursors were kept in stainless steel tanks at room‐temperature (RT) under nitrogen pressure. The injection into the reaction chamber was made through individual KEMSTREAM Vapbox 300 injection heads. The depositions were performed at a pressure of 160 Pa; 300 sccm nitrogen was used as carrier gas in the injector mixing chamber, and 140 sccm argon was introduced in the reaction chamber. The process temperatures of 100, 150, 180, and 150 °C were used on the injection heads, gas lines, vapor lines, and substrate, respectively. The purge time was maintained at 10 s, while the precursor's mass flow was variated as presented in Table 1.

The deposition of HfO_2_ has been carried out simultaneously on both (*i*) simple commercial Si++ covered with 50 nm thick SiO_2_ and (*ii*) *c*‐axis textured 1200 nm thick AlN coated substrates. The AlN coating was prepared by radio‐frequency magnetron sputtering (RF‐MS). The AlN film, crystallized in a hexagonal system, elicited texturing along *c*‐axis (Figure S10, Supporting Information), as reported in Besleaga et al.^[^ ^59^ ^]^.

### Characterization

The transmission electron microscopy (TEM) analyses were performed on JEM‐ARM 200F and JEOL 2100 microscopes, operated at 200 kV. The cross‐section TEM specimens were prepared by mechanical polishing down to ≈30 µm, followed by ion milling in a Gatan PIPS machine at 4 kV accelerating voltage and 7° incidence angle. Low‐voltage (2 kV) milling was used as the final ion polishing stage in order to reduce the amorphous surface layer enveloping the specimen.

The crystalline status of the films was investigated by grazing incidence X‐ray diffraction (GIXRD, incidence angle = 2°), using a Rigaku SmartLab 3 kW system with CuK_α_ radiation (λ = 1.5418 Å). The GIXRD patterns were acquired in the angular (2θ) range of 20–37° with a step size of 0.02° and a scan speed of 0.3°/min.

The vibrational structure of the films was studied by FTIR spectroscopy in attenuated total reflectance (ATR) mode. In this scope, a Jasco 6800‐FV‐BB spectrometer (Jasco Corporation, Tokyo, Japan) equipped with an ATR PRO670H attachment with bulk diamond crystal, was used. The FTIR‐ATR spectra were recorded under vacuum in the spectral range of 850–100 cm^−1^, at a resolution of 4 cm^−1^, and represent the average of a minimum 64 individual scans.

The deconvolution of the XRD patterns and FTIR spectra was performed using the Fityk 0.9.4 analysis program, employing Gaussian functions.

The XPS was performed with a SPECS GmbH equipment having a PHOIBOS 150 analyzer. The measurements were performed using an XR 50 M monochromatic X‐ray source set at 300 W with Al K_α_ (1486.7 eV) radiation. The analyzer was operated at a pass energy of 20 eV for the acquisition of high‐resolution scans of relevant core electron levels. The sample neutralization during the measurements was achieved using a Specs FG15/40 flood gun. The fitting of the XPS spectra was performed using the spectral data processor program, Voigt functions, and tabulated Scofield sensitivity factors.

For the electrical measurements, Ti/Au top electrodes with an area of 0.44 mm^2^ were deposited by RF‐MS. The Capacitance‐Voltage (C–V) and Conductance‐Voltage (G–V) measurements were performed in a Lakeshore cryostation with micro‐manipulated arms, at RT, in air and dark. The data were recorded using a Hioki 3536 LCR bridge connected to a Keithley 6517 electrometer with an incorporated dc voltage source. The measurements are performed using an AC signal with a frequency of 100 kHz and an amplitude of 0.05 V.

The low temperature capacitance measurements were performed with a Hioky impedance analyzer IM3570, with the samples kept in a Lake Shore cryostat, under a 10^−4^ Pa vacuum. The same samples were used for the capacitance and pyroelectric effect measurements.

The pyroelectric properties were explored using a SR 830 DSP lock‐in amplifier and a J‐FET type impedance converter (see details in Supporting Information,^[^ ^60^ ^]^ Figure S11, Supporting Information). The IR source was a laser diode of 40 mW at 800 nm. The beam was modulated electronically, using a signal generator from Tektronix, model AFG 3052C. A device electrode area of 0.485 mm^2^ was employed.

## Conflict of Interest

The authors declare no conflict of interest.

## Supporting information



Supporting Information

## Data Availability

The data that support the findings of this study are available from the corresponding author upon reasonable request.
