# Supplementary material for: Electronic–Structural Phase Correlations in Oxygen‐Deficient Hafnia Nanocrystals
Source: Small. 2025 Nov 20;22(2):e08888. doi: 10.1002/smll.202508888 (PMC12781617; doi:10.1002/smll.202508888)
Supplement: Supplementary file 1 — Supporting Information [file SMLL-22-e08888-s001.docx]

Supporting Information

**Electronic–Structural Phase Correlations in Oxygen-Deficient Hafnia Nanocrystals**

*Cristina Besleaga*, Mihaela Botea, Catalin C. Negrila, Andrei Kuncser, Cosmin M. Istrate, Andrei Nitescu, George E. Stan, Swayam P. Sahoo, Bertrand Vilquin, Lucian Pintilie*

Figure S1:


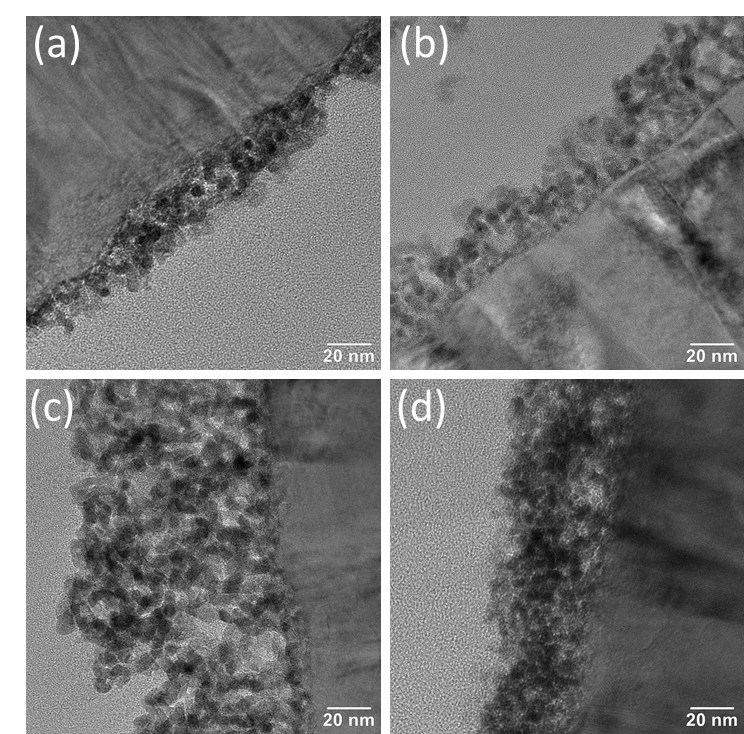


**Fig. S1.** *TEM images characteristic to the (a) HfO_2_-0.6, (b) (Hf,Zr)O_2_-0.6, (c)* *HfO_2_-2.2, and (d) (Hf,Zr)O_2_-2.2 layers.*

Figure S2:


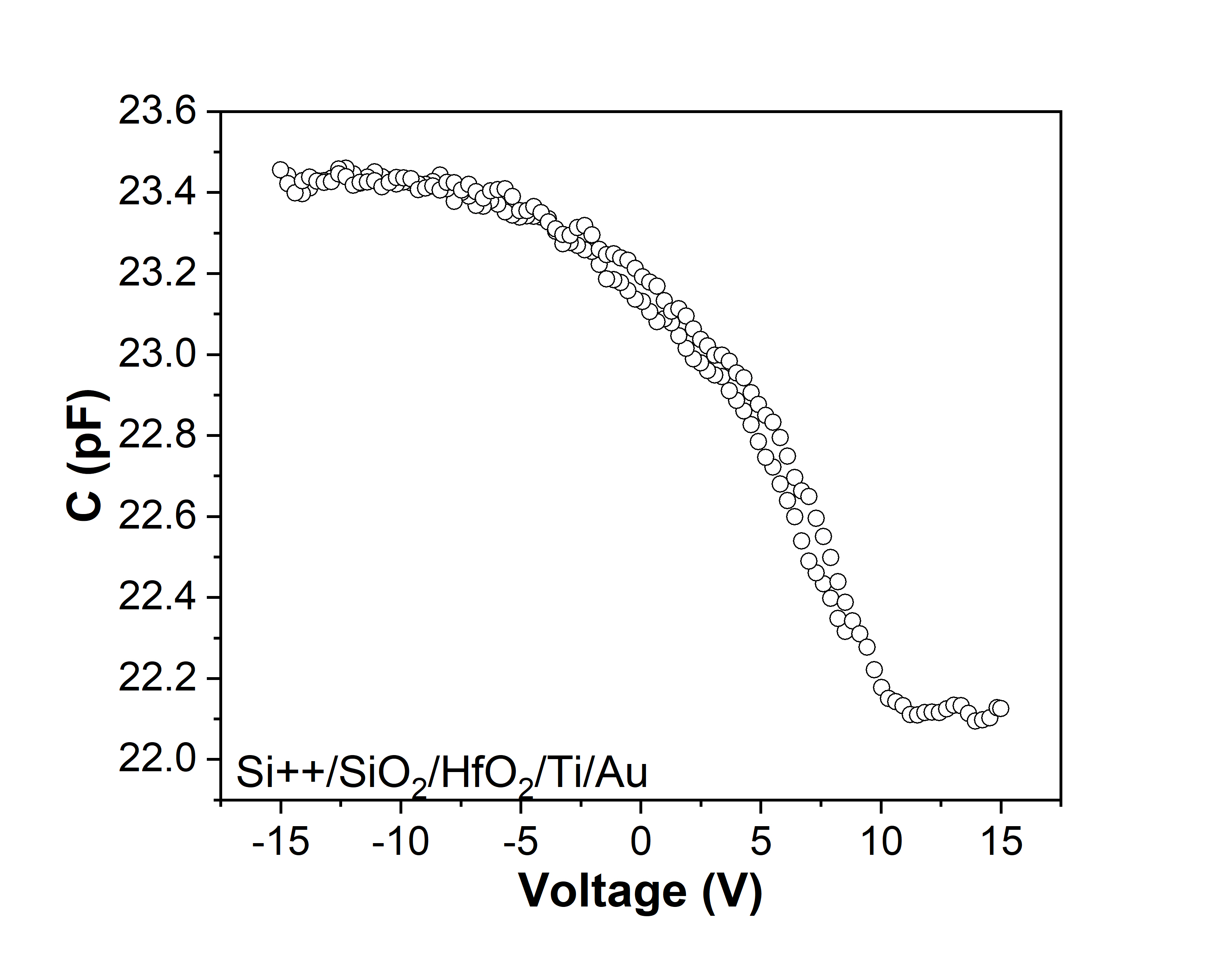


**Fig. S2**. *Capacitance vs. Voltage characteristics at RT for Si++/SiO_2_/ HfO_2_-2.2 device; 100 kHz, 0.5 V AC signal.*

**Table S1:** *The 2θ of the XRD prominent peak for different orthorhombic phases, polar and non-polar, tetragonal and cubic phases:*

| **Phase** | **Space group** | **Type** | **XRD prominent peak** |
| --- | --- | --- | --- |
| Orthorhombic | 61/Pbca | Nonpolar ^[9]^ | 30.87 *# ICDD-PDF4 no. 04-011-8819*  30.35° *# ICDD-PDF4 no. 01-081-0028* |
| Orthorhombic | 29/Pca21 | Polar ^[9]^ | 29.9° *# ICDD-PDF4 no. 04-005-5597* |
| Orthorhombic | 62/Pnma | Antipolar ^[9]^ | 31.9° *# ICDD-PDF4 no. 01-078-5758* |
| Orthorhombic | 62/Pmnb |  | 34.47° *# ICDD-PDF4 no. 01-089-6226*  35.73° *# ICDD-PDF4 no. 01-089-6225* |
| Orthorhombic | 62/Pnam |  | 32.04° *# ICDD-PDF4 no. 01-087-2106* |
| Orthorhombic | 57/Pbcm |  | 30.36° *# ICDD-PDF4 no. 01-070-2832* |
| Tetragonal | 137/P4_2_nmc | Nonpolar ^[9]^ | 29.3° *# ICDD-PDF4 no. 04-002-5353*  30.3° *# ICDD-PDF4 no. 04-011-8820* |
| Cubic | 225/F3m3 | Nonpolar^[9]^ | 30.57° *# ICDD-PDF4 no. 04-011-9018*  30.36° *# ICDD-PDF4 no. 00-053-0560* |

Figure S3:

*
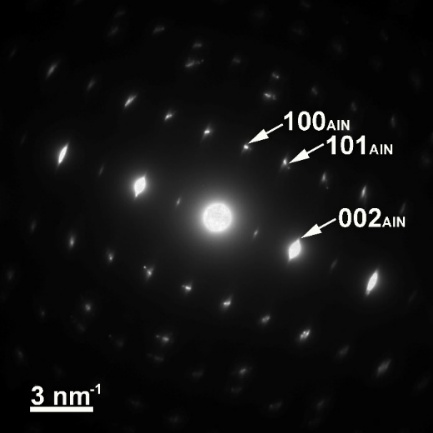

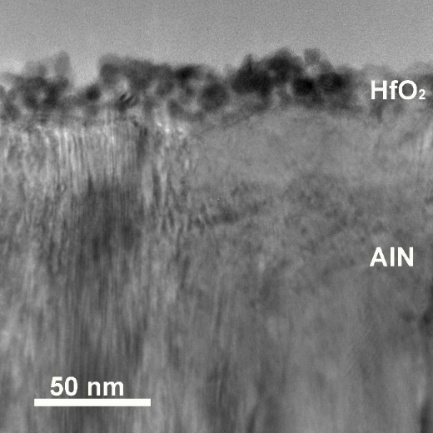

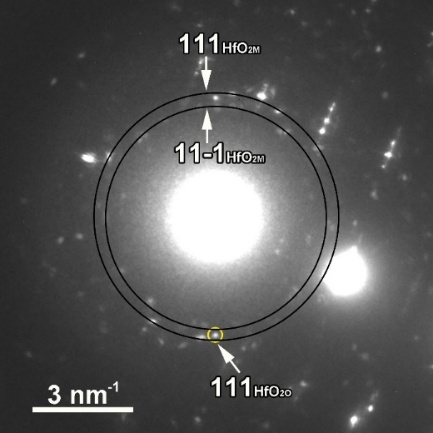
*

**Fig S3** *. Low-magnification TEM image showing the spatial distribution and morphology of HfO₂ nanocrystallites deposited on AlN. The first electron diffraction pattern was obtained using a selected-area aperture positioned over a region containing only AlN, while the second diffraction pattern was acquired with the smallest available aperture in order to capture several HfO₂ nanocrystallites.*

Figure S4:

*
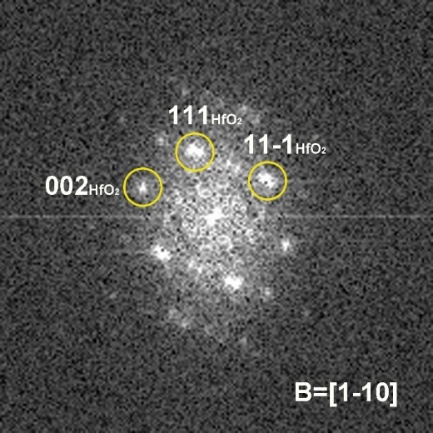

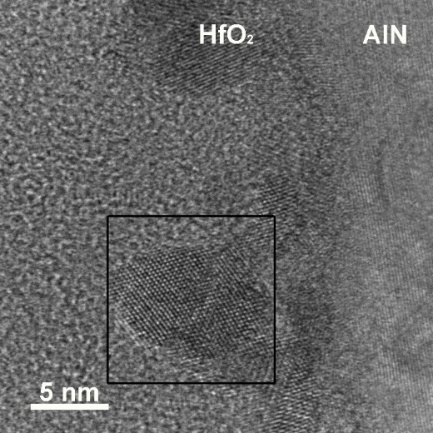

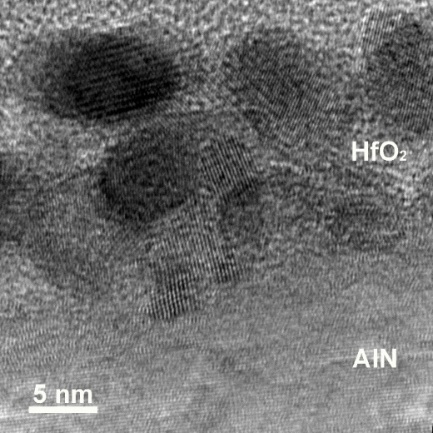

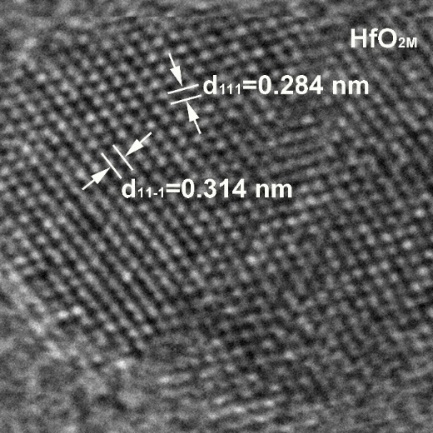

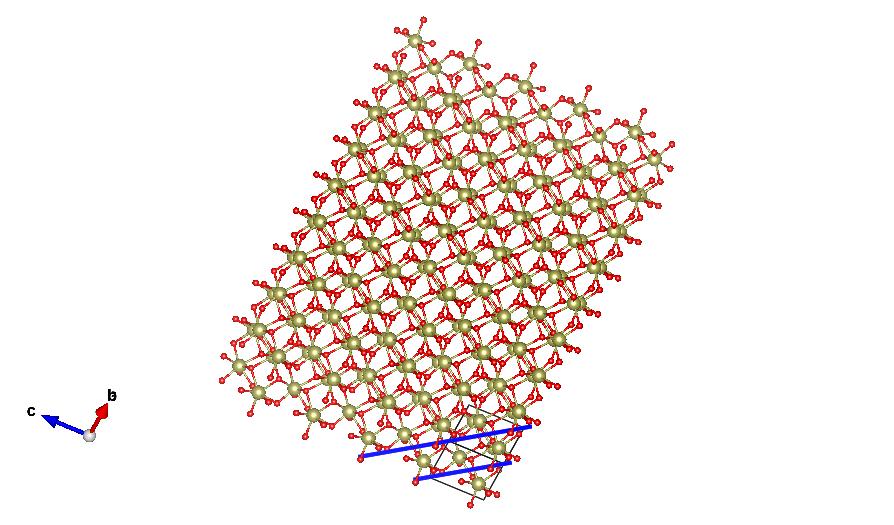
*

**e**

**d**

**c**

**b**

**a**

**d**

**c**

**b**

**Fig S4.** *(a), (b) High-magnification images of HfO₂ nanocrystallites deposited on AlN. (c) Enlarged image of the area outlined by the black square in Figure (b), showing an HfO₂ crystallite where the interplanar spacings corresponding to the (111) and (11-1) planes of monoclinic HfO₂ were measured. (d) Fourier transform obtained from the area marked by the black square, containing only the HfO₂ crystallite. (e) Atomic structural model of HfO₂ along the zone axis B = [1-10], determined based on the measurements performed on the Fourier transform.*

To identify the crystalline structure, we performed electron diffraction analysis. In Figure S3, the low-magnification TEM image shows the morphology and spatial distribution of the HfO_2_ nanocrystallites. These exhibit a relatively spherical shape, with sizes ranging from 5 to 12 nm, showing a tendency to agglomerate and adhere to one another. However, regions were identified where individual nanocrystallites could be clearly distinguished, allowing the boundaries between them to be marked.

Two electron diffraction patterns were acquired, the first one corresponding to an area containing only AlN. Based on the measurements performed, several peaks were successfully indexed, as highlighted in Figure S3. These measurements led to the conclusion that AlN has a hexagonal crystalline phase with space group P6₃mc. The corresponding diffraction pattern also contains several spots forbidden by the structure factor, mainly due to double diffraction effects.

Using the smallest possible selected-area aperture, the electron diffraction pattern shown in Figure S3 (right) contains peaks originating from both the HfO2 nanocrystallites and the AlN layer, making it impossible to isolate a region containing only HfO2. Based on the diffraction pattern measurements, two circles were drawn, corresponding in real space to interplanar spacings of 0.284 nm for the upper circle and 0.314 nm for the lower one. All peaks aligned on the upper circle can be attributed to the (111) planes of the monoclinic structure, while those on the lower circle correspond to the (11-1) planes of monoclinic HfO_2_.

These results indicate that most of the HfO_2_ nanocrystallites possess a monoclinic structure. Indexing was performed using the monoclinic HfO₂ structure with space group P2₁/c and lattice parameters a = 0.5233 nm, b = 0.5268 nm, and c = 0.5414 nm. However, a set of peaks was identified between the two circles, corresponding to a spacing of 0.296 nm. These could be assigned either to the (111) planes of orthorhombic HfO_2_ or to the (101) planes of the tetragonal HfO_2_ structure, although determining the exact space group is practically impossible. At this stage, we can conclude that the monoclinic phase is predominant, with a few nanocrystallites possibly exhibiting a tetragonal or orthorhombic structure.

Furthermore, Figures S4-a,b show two high-resolution images illustrating the morphology and crystallinity of the HfO_2_ nanocrystallites. Figure S4 a reveals their tendency to agglomerate and their approximately spherical shape. To confirm the analysis obtained from the electron diffraction pattern, we selected an HfO_2_ nanocrystallite that was perfectly oriented with respect to the electron beam; an example is shown in Figure S4-b,c presents a magnified image of the HfO_2_ nanocrystallite, where atomic planes can be clearly observed and their interplanar spacings measured.

The Fourier transform shown in Figure S4-d was obtained from the area marked by the black square in Figure S4-b and contains peaks corresponding to the atomic planes visible in the high-resolution image. Based on the Fourier transform analysis, several peaks were identified and assigned to the (11-1), (111), and (002) planes of the monoclinic HfO_2_ structure. The measured interplanar distances of 0.285 nm for (111) and 0.314 nm for (11-1) planes are strong indicators of the monoclinic structure, since in the orthorhombic/tetragonal structures these distances are equal. Figure S4-e shows the atomic structural model of monoclinic HfO_2_ along the [1-10] zone axis, which is consistent with the high-resolution microscopy images.

**FTIR vibration modes specific to hafnia phases:**

According to [8-10], the FTIR vibration mode at:

~**122 cm^-1^** can be ascribed to A_1_(TO) – polar orthorhombic Pca21; B_1u_ – antipolar orthorhombic Pnma or to E_u_(TO) tetragonal;

~**266 cm^-1^** can be ascribed to A_1_(TO) – polar orthorhombic Pca2; T_1u_(TO) – cubic; E_u_(LO) – tetragonal;

~**322 cm^-1^** can be ascribed to A_2u_(TO) – tetragonal;

**~350 cm^-1^** can be ascribed to B_u_(TO) – monoclinic; B_2u_(LO) – antipolar orthorhombic Pbca; B_1_(LO) – polar orthorhombic Pca21; B_1_(LO) – orthorhombic Pmn21;

**~394 cm^-1^** can be ascribed to A_u_(LO) – monoclinic; A_1_(LO) – polar orthorhombic Pca21; B_2g_ – antipolar orthorhombic Pnma;

**~417 cm^-1^** can be ascribed to A_u_(TO) – monoclinic; B_1u_(LO) – antipolar orthorhombic Pbca;

**~472 cm^-1^** can be ascribed to A_1_(TO) – polar orthorhombic Pca21;

**~485 cm^-1^** can be ascribed to A_u_(LO) – monoclinic; B_1u_(TO) – antipolar orthorhombic Pbca; B_2g_ – antipolar orthorhombic Pnma;

**~514 cm^-1^** can be ascribed to B_u_(LO) – monoclinic; B_2_(LO) – polar orthorhombic Pca21;

**~552 cm^-1^** can be ascribed to B_3u_(LO) – antipolar orthorhombic Pbca; B_3u_(TO) – antipolar orthorhombic Pnma;

**~565 cm^-1^** can be ascribed to B_2_(TO) – polar orthorhombic Pca21;

**~617 cm^-1^** can be ascribed to A_u_(TO) – monoclinic; B_1_(LO) – polar orthorhombic Pca21;

**~760 cm^-1^** can be ascribed to B_2g_ – antipolar orthorhombic Pnma.

Figure S5:


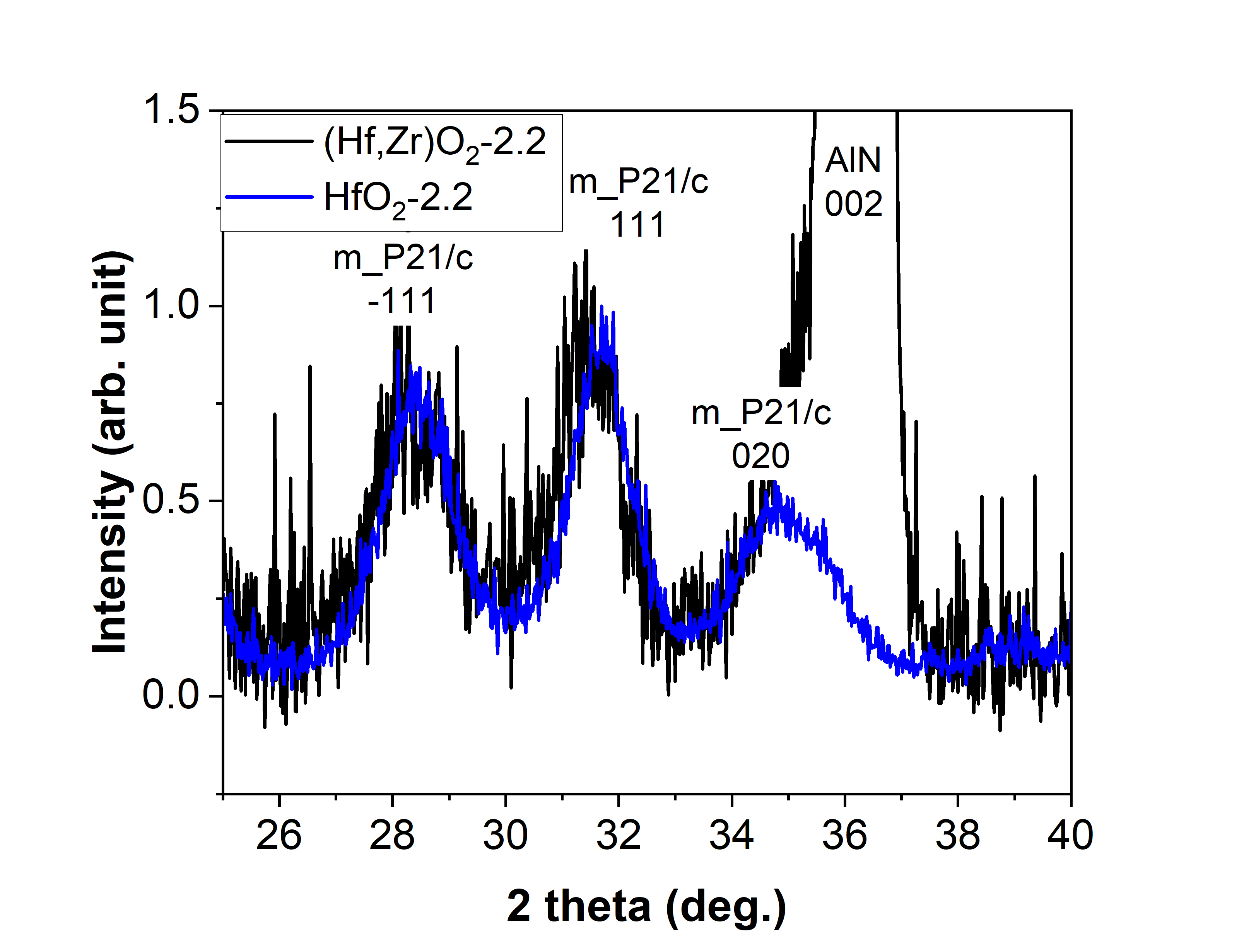


**Fig. S5**: *GIXRD patterns of the* (Hf,Zr)O_2_-2.2 and *HfO_2_-2.2 layers on AlN*

Methodology for HZO thin films obtaining by RF-magnetron sputtering:

The n-type Si(100) substrates were subjected to a standard cleaning protocol involving sequential rinsing with acetone and ethanol to eliminate organic surface contaminants. To remove the native silicon oxide layer, a buffered oxide etch (BOE) process was employed. HZO thin film was deposited on a silicon substrate at room temperature via radio frequency (RF) magnetron sputtering using a stoichiometric Hf_0.5_Zr_0.5_O_2_ ceramic target in an Alliance Concept AC-450 sputtering system. The deposition was carried out in a pure argon (Ar) ambient at a working pressure of 5 × 10^-2^ mbar. The as-deposited HZO films were amorphous, and the crystallization was induced through rapid thermal annealing (RTA) at 450 °C for 30 seconds in a nitrogen (N_2_) environment [47].

| **RF Magnetron Sputtering film growth parameters:** | | |
| --- | --- | --- |
| Target-substrate distance | 8 cm | |
| Base pressure(P) | 5*×*10*^−^*^8^ < P < 5*×*10*^−^*^7^ mbar | |
| Deposited elements | HZO | |
| Substrate | n^+^Si(100) | |
| Target | Hf_0.5_Zr_0.5_O_2_ | |
| Target RF power | 100W | |
| Gas | Ar 50 sccm | |
| Working pressure (mbar) | 5*×*10*^2^* | |
| Substrate temperature | Room Temperature | |
| Deposition speed (nm/min) | ̴ 4 | |
| Rapid thermal annealing (RTA) conditions of HZO | | |
| Temperature (°C) | Atmosphere | Time (s) |
| 450 | N_2_ | 30 |

Figure S6:


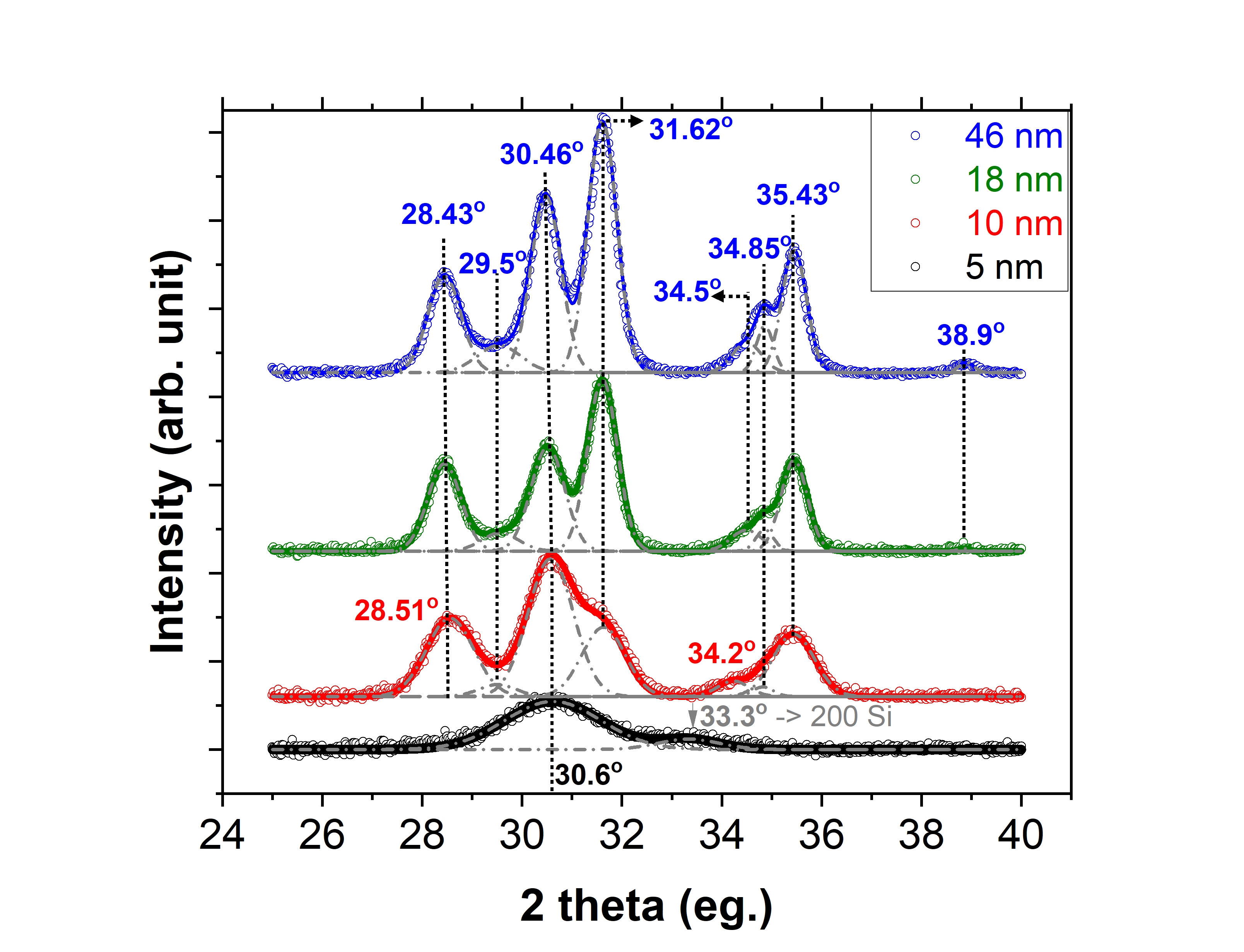


**Fig. S6**: *GIXRD patterns of the* (Hf,Zr)O_2_ *layers deposited by RF-magnetron sputtering on (100) silicon; film thickness: 5 nm (black), 10 nm (red), 18 nm (green) and 46 nm (blue)*

Figure S7:


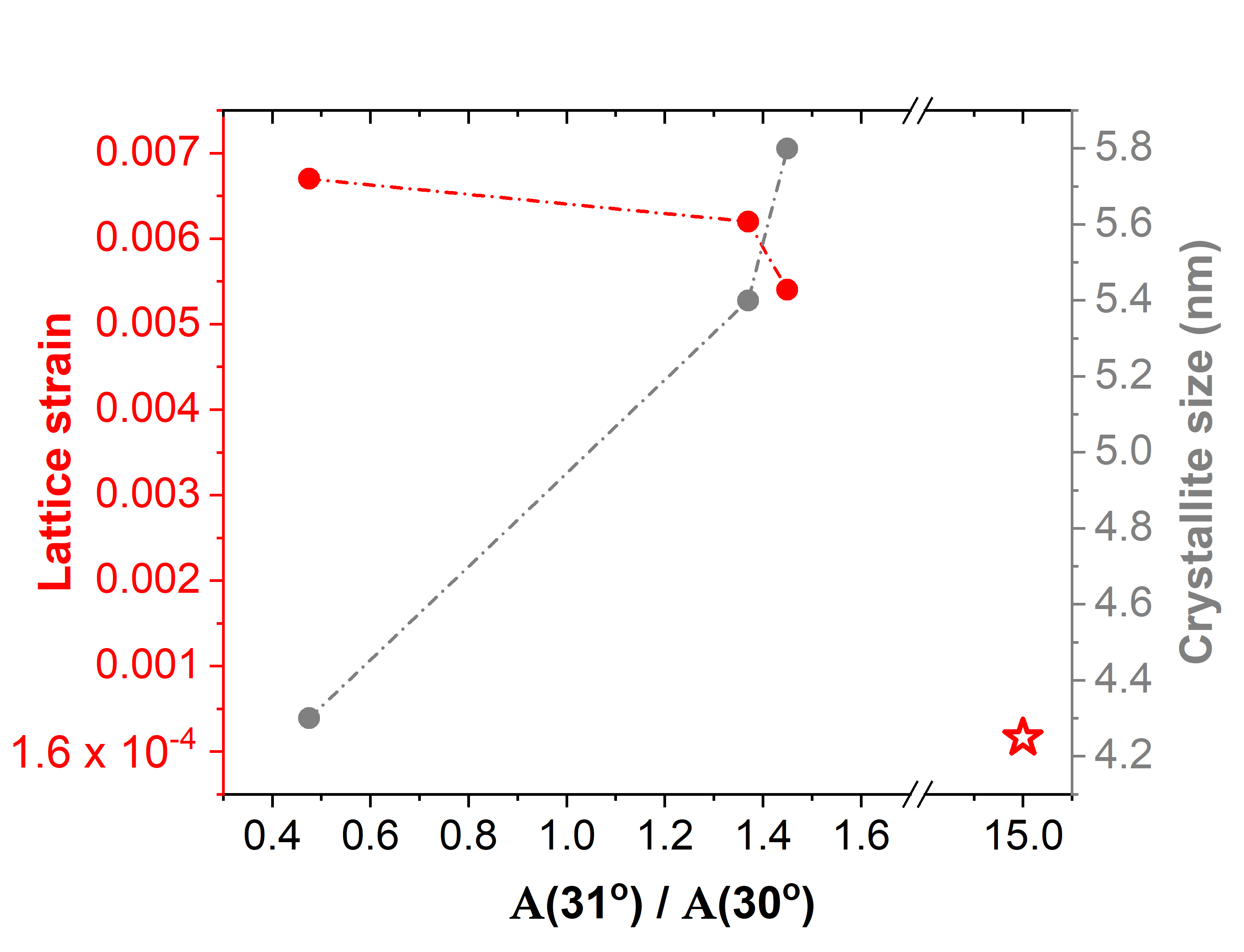


**Fig. S7:** *Lattice strain (red) and crystallite size (gray) for RF-sputtered (Hf,Zr)O_2_ (dots) and nano-grains (Hf,Zr)O_2_-2.2 (star) layers vs. the ratio between the integral areas of monoclinic (111) peak and the peak at ~ 30°*

Figure S8, FTIR for *sputtered (Hf,Zr)O_2_* films:


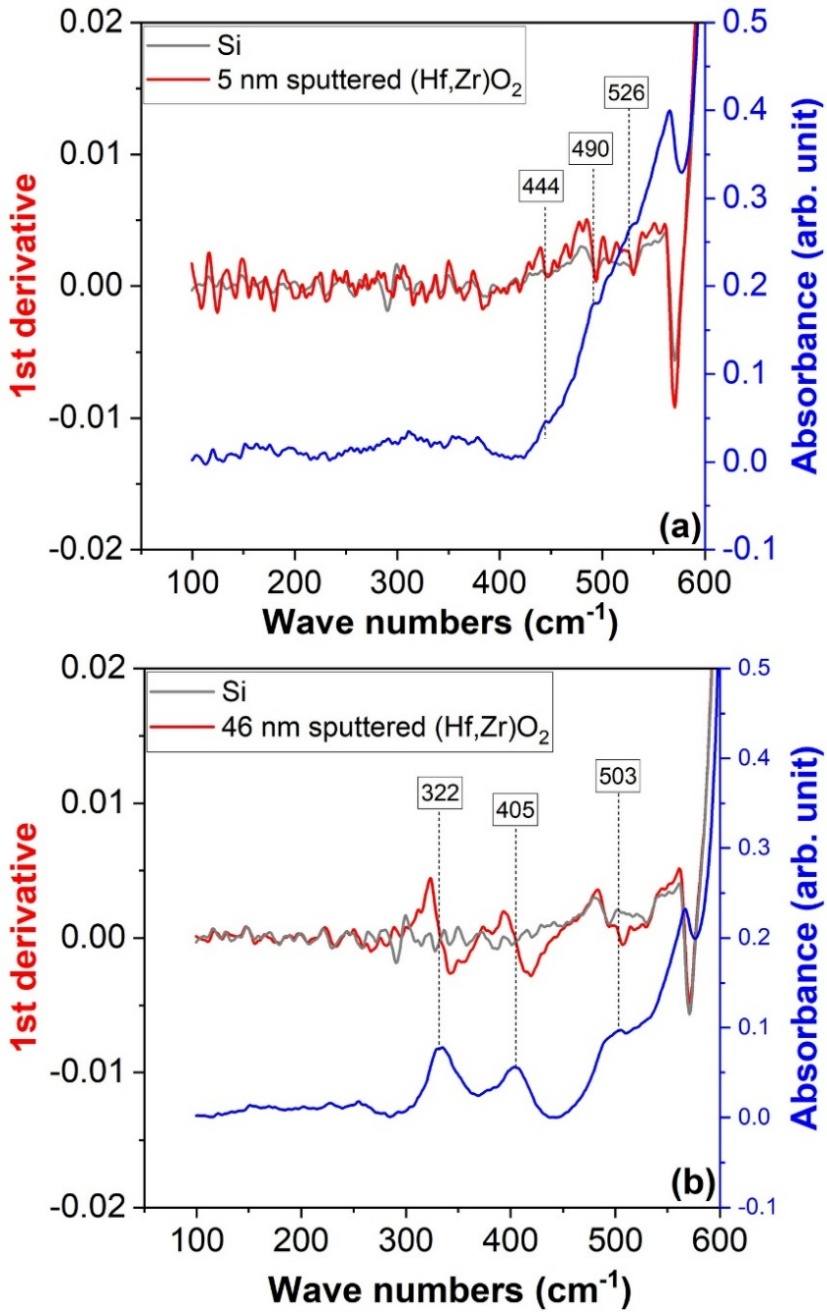


**Fig. S8**: *The FTIR-ATR spectra of the rf-magnetron sputtered (Hf,Zr)O_2_ (a) 5 nm thick and (b) 46 nm thick; absorbance (blue line) and first derivative (red line for HfO_2_-2.2 and gray line for Si substrate).*

According to [8–10], the FTIR vibration mode at:

~**322 cm^-1^** can be ascribed to A_2u_(TO) – tetragonal.

~**405 cm^-1^** can be ascribed to Bu(TO) – monoclinic; B_2u_(LO) – orthorhombic-antipolar Pbca; B_2_(TO) – orthorhombic polar Pca21;

~**444** **cm^-1^** can be ascribed to B_1u_(LO) – orthorhombic antipolar Pnma;

~**490 cm^-1^** can be ascribed to B_1u_(TO) – antipolar orthorhombic Pbca; B1(TO) – polar orthorhombic Pca21; B2(TO) –orthorhombic Pmn21; B_2g_ – antipolar orthorhombic Pnma;

~ **503** **cm^-1^** can be ascribed to Au(TO) – monoclinic; B_2u_(LO) – orthorhombic antipolar Pbca; B1(TO) – polar orthorhombic Pca21;

~ 526 **cm^-1^** can be ascribed to Bu(TO) – monoclinic; B3u – antipolar orthorhombic Pnma (ZrO_2_).

Figure S9


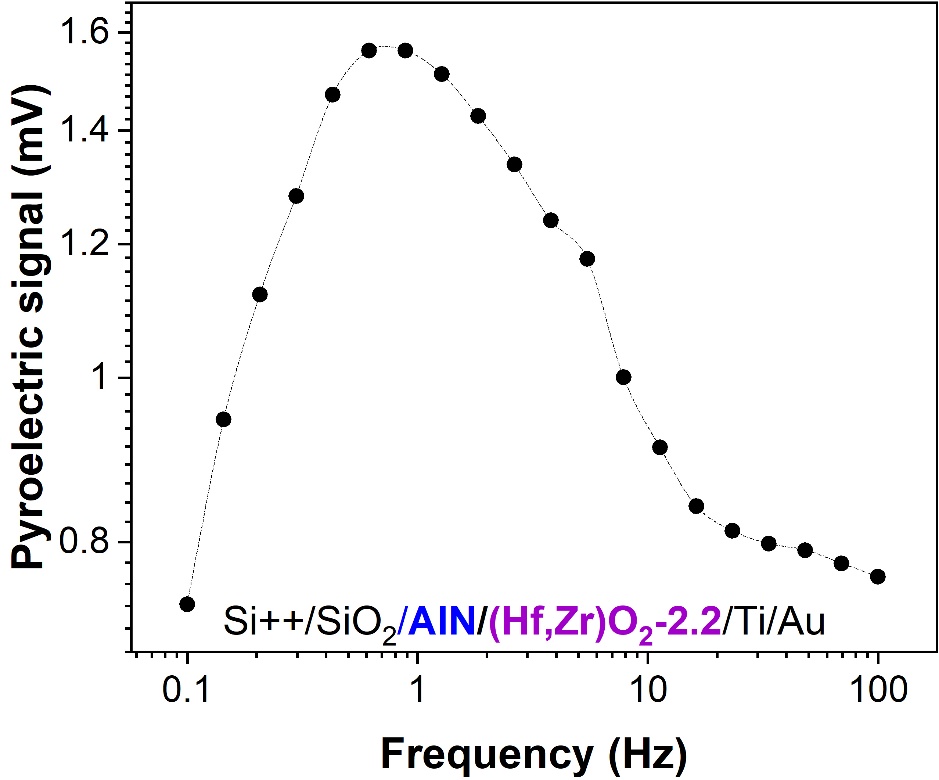


**Fig. S9**: *The frequency dependence of the pyroelectric signal for Si++/SiO_2_/AlN/(Hf,Zr)O_2_-2.2 device*

Figure S10:


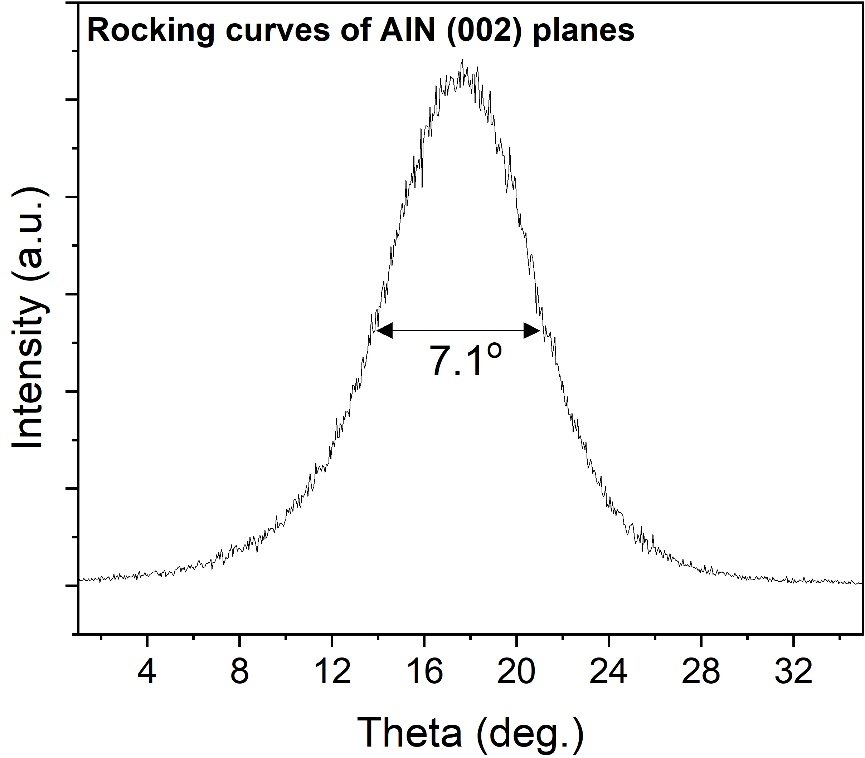


**Fig. S10:** *The (002) peak the x-ray rocking curve measurements of AlN thin films*

**Pyroelectric setup:**

The pyroelectric signal generated under illumination with a modulated IR beam. In this way are avoided detrimental influences from thermally stimulated and leakage currents.

Pyroelectric detectors can operate in current or voltage mode [60]. We used the voltage mode, where the pyroelectric element is connected to the gate electrode of a field effect transistor. The frequency dependence of the pyroelectric signal was recorded using as IR source a laser diode from ThorLabs (model Thorlabs M9_808_0150, wavelength 808 nm, maximum power 150 mW). The laser beam was electronically modulated using a signal generator model Tektronix AFG 3052C, while the pyroelectric signal was read using a lock-in amplifier model Stanford Research - SR 830 DSP.


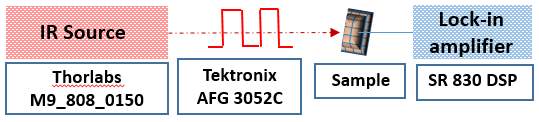


**Fig. S11.** *Setup schematic for pyroelectric measurements*

The Si++ substrate was thermally shunted using a metallic heat sink attached to the substrate backside and edges with highly thermal conductive silver paste.

Figure S12


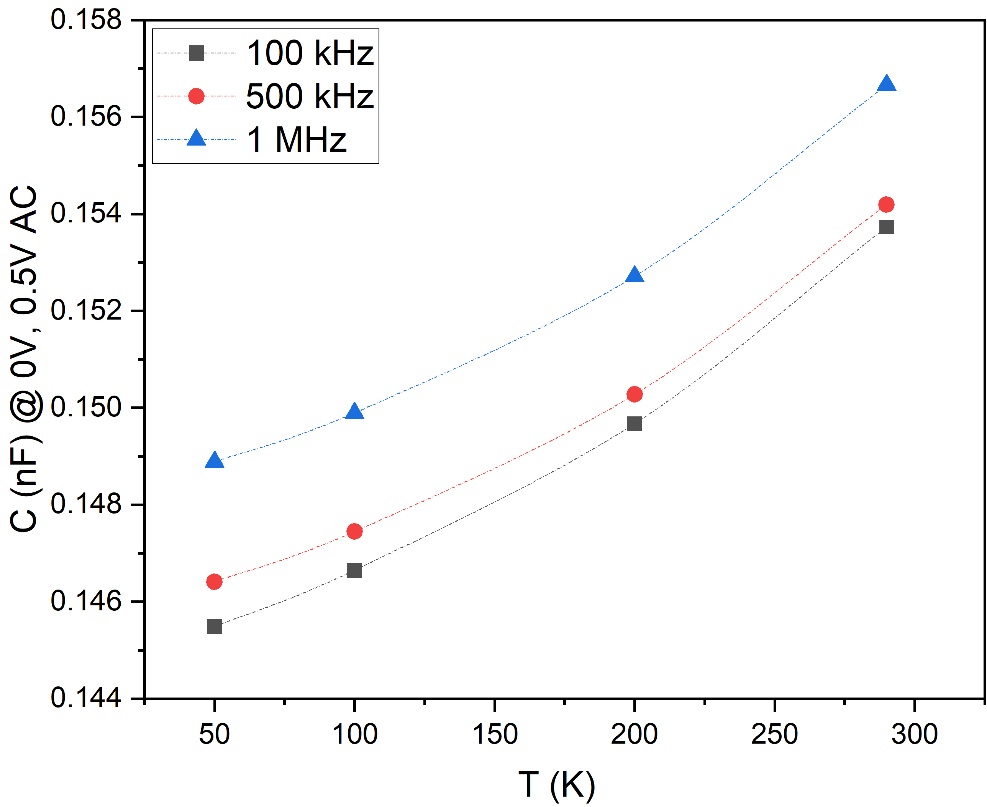


**Fig. S12.** *Capacitance dispersion at various temperatures for Si++/SiO2/HfO_2_-2.2 device*
